# Maslinic Acid Attenuates Ischemia/Reperfusion-Induced Acute Kidney Injury by Suppressing Inflammation and Apoptosis Through Inhibiting NF-κB and MAPK Signaling Pathway

**DOI:** 10.3389/fphar.2022.807452

**Published:** 2022-04-12

**Authors:** Wenjuan Sun, Hong Sang Choi, Chang Seong Kim, Eun Hui Bae, Seong Kwon Ma, Soo Wan Kim

**Affiliations:** Department of Internal Medicine, Chonnam National University Medical School, Gwangju, South Korea

**Keywords:** ischemia-reperfusion injury, maslinic acid, NF-κB, MAPK, kidney diseases

## Abstract

Inflammation and apoptosis are the major contributors to the mechanisms of acute kidney injury (AKI) due to renal ischemia-reperfusion injury (IRI). Maslinic acid (MA), a pentacyclic triterpene acid mostly found in dietary plants, the current study was to demonstrate the renoprotective effect of MA on IRI-induced AKI, and to investigate the role of inflammation and apoptosis-related signaling pathways as a molecular mechanism. C57BL/6J mice were subjected to IRI for 72 h, and MA was daily administered by intraperitoneal injection during this period. In parallel, rat renal proximal tubule cells (NRK52E) were prophylactically treated with MA and then exposed to hydrogen peroxide (H_2_O_2_). MA treatment significantly inhibited the mRNA expression of interleukin (IL-1β), tumor necrosis factor-α (TGF-α), monocyte chemoattractant protein-1 (MCP-1), and intercellular adhesion molecule-1(ICAM-1). Also, MA reduced the expression of Bax/Bcl2 ratio and cleaved caspase-3. In NRK52 cells, MA inhibited the IκBα degradation, blocked NF-κB/p65 phosphorylation, and nuclear translocation. The phosphorylation of ERK, JNK, and p38 was attenuated by MA in IRI-induced kidney injury and H_2_O_2_-stimulated NRK52 cells. The expression levels of IL-1β, MCP-1, and ICAM-1 were upregulated in H_2_O_2_-stimulated NRK52E cells, which was attenuated by NF-κB inhibitor. H_2_O_2_ treatment increased the Bax/Bcl2 ratio and cleaved caspase-3 in NRK52E cells, which was counteracted by MAPK inhibitors. Together, our data demonstrate that MA suppresses IR-induced AKI injury through NF-κB and MAPK signaling pathways and that MA is a promising agent in the treatment of kidney diseases.

## Introduction

Acute kidney injury (AKI) is a common clinical emergency and critical illness. In particular, kidney transplantation and kidney surgery are common susceptibility factors (Boratyńska et al., 2004; Cooper and Wiseman, 2013; Andreucci et al., 2014). The main cause of AKI, renal ischemia/reperfusion injury (IRI), is clinically related to the significant morbidity and mortality of patients with AKI, which may lead to a prolonged hospital stay and irreversible damage of the kidney (Dong et al., 2019).

The reduction in blood flow caused by the above-mentioned factors can lead to hypoxia in the kidney tissue, and the rapid restoration of blood supply can lead to IRI (Dorweiler et al., 2007; Bonventre and Yang, 2011). During this period, it leads to various cellular responses, such as massive production of reactive oxygen species (ROS), calcium overload, and other direct or indirect activation of apoptotic signaling pathways, initiating infiltration of inflammatory factors and the release of reactive mediators, ultimately leading to structural damage and long-term tissue injury (Forbes et al., 2000; Han and Lee, 2019). Hydrogen peroxide (H_2_O_2_), which is produced by enzymatic catalysis or spontaneous decomposition of superoxide anions, is more stable than other ROS members and is membrane-permeable (Phaniendra et al., 2015). Therefore, H_2_O_2_ is considered a key mediator of renal tubular injury in various pathological conditions, especially in the cascade of cellular responses induced by renal IRI (Kim et al., 2009). Correspondingly, exogenous H_2_O_2_ has been widely used to induce ROS-mediated oxidative damage in renal tubular epithelial cells.

Inflammation is important for the appearance, deterioration, and prognosis of IRI (Jo et al., 2006; Thurman, 2007; Dellepiane et al., 2016). It involves endothelial cells and renal tubular cells releasing inflammatory mediators, inflammatory cell infiltration, and the effect of toxic molecules on the renal tubules (Bonventre and Yang, 2011). It is worth noting that when renal tissue is reperfused after ischemia, it may cause the kidney to produce a large amount of ROS, as we mentioned before. H_2_O_2_ was significantly increased in small arteries and renal cortex tissues isolated after IRI (Huang et al., 2016). Renal cells activated by H_2_O_2_ could produce inflammatory mediators, free radicals, and other destructive substances, which will eventually lead to significant renal dysfunction (Mittal et al., 2014; Granger and Kvietys, 2015; Gunawardena et al., 2019).

Another important process related to IRI is apoptosis based on the overwhelming evidence. Cells trigger specific signaling pathways due to inflammation or oxidative stress to cause cell death (Devarajan, 2006). H_2_O_2_ could damage renal tubular epithelial cells by activating apoptosis pathways (such as the intrinsic cell death pathway mediated by mitochondria) and accelerating the development of kidney diseases (Linkermann et al., 2014). Specifically, when apoptosis occurs, the outer mitochondrial membrane is destroyed, and the apoptosis-inducing genes such as TNF-α, Bax, and caspase, which regulate mitochondria, are highly expressed, and the expression of the important anti-apoptotic gene Bcl-2 in the body is decreased simultaneously (Havasi and Borkan, 2011; Yang et al., 2018).

The molecular structure of MA is a pentacyclic triterpene. The compound is derived from natural leaves and fruits of various plants, such as olive and oleander (Choudhary et al., 2021). It has been demonstrated that MA has a wide range of pharmacological effects, including antiproliferative activity, antitumor activity, antioxidant effect, and anti-inflammatory activity (Juan et al., 2008; Reyes-Zurita et al., 2009; Li et al., 2011; Li et al., 2017). However, so far, there is rare research on the beneficial role of MA on IRI-induced AKI.

## Materials and Methods

### Chemicals and Reagents

MA (M6699) was purchased from Sigma-Aldrich (Louis Mo. United States). Anti-p-(NF)-κB-P65 (SC-33020), anti-(NF)-κB-P65 (SC-372); anti-IκBα (SC-1643); anti-Nrf2 (SC-722); anti-ATP5A (SC-136178) and anti-β-actin (SC-47778) were from Santa Cruz Biotechnology (Santa Cruz, CA,United States); anti-GAPDH (AM4300; Ambion, Austin, TX, United States); anti-Lamin B antibody (ab16048, Abcam, Cambridge, United Kingdom). Anti-ERK1/2 (#9102S); anti-phosphorylated ERK1/2 (p-ERK1/2) (#9101S); anti-JNK (#9252S); anti-phosphorylated JNK (p-JNK) (#9251S); anti-P38 (#9212S); anti-phosphorylated P38 (p-P38) (#9215S); anti-Bcl-2 (#3498S); anti-Bax (#2772S); anti-Cleaved caspase3 (#9661S) and anti-caspase3 (#9662S) from Cell Signaling Technology (Danvers, MA, United States); ERK1/2 inhibitor PD (#513000), specific JNK inhibitor SP (#420119), p38 MAPK inhibitor SB (#559387) were from Calbiochem (San Diego, CA), Bay, an NF-κB inhibitor (Cay-10010266) was purchased from BioMol GmbH (Hamburg, Germany).

### Animal Experiments

Six-week-old male C57BL/6 mice (18–22 g) were obtained from Samtako (Osan, South Korea). We randomly divided the mice into three groups: the sham-operated group, the IRI group, and the MA-treated IRI group (IRI + MA). A midventral incision was used to expose the abdominal cavity. Mice were anesthetized with 2% isoflurane and the renal pedicles were clamped with micro clamps (ROBOZ, Gaithersburg, United States) for 30 min to cause ischemia in the kidneys. They were then placed on a temperature control table (37.5°C) to maintain body temperature. After 72 h of reperfusion, all mice were sacrificed. Mice in the IRI + MA group were injected intraperitoneally with MA at a dose of 20 mg/kg (dissolved in 20 µl DMSO) pre-treatment to the surgery procedure based on our previous report (Sun et al., 2021). MA was injected intraperitoneally in two protocols: In protocol 1 (n = 8 in each group), the prescribed dose of 20 mg/kg was injected 24 h before the ischemic surgery and the same dose was injected every day thereafter until the day before execution; in protocol 2 (n = 6 in each group), the same dose was injected once 1 h before the ischemic surgery. The sham group underwent the same protocol as the IRI group, except that the clamp was not used. The left kidney was quickly extracted and prepared for the following experiment, and the right kidney was frozen at -80 °C.

### Cell Culture and Treatment

Rat proximal tubular epithelial (NRK52E) cells (American Type Culture Collection, Manassas, VA, United States) were incubated at 37°C under a 5% CO_2_ atmosphere in high-glucose Dulbecco’s modified Eagle’s medium (DMEM; Welgene, Daegu, South Korea) with 5% FBS and 1% streptomycin/penicillin. Cells were seeded on 60 mm plates at 70–80% confluence and then treated with MA or vehicle-alone (DMSO) 2 h after being starved with no FBS media. Then the cells were incubated with H_2_O_2_ (Rocky Hill, NJ, United States) for a certain duration of time. MA was dissolved in dimethyl sulfoxide (DMSO) to make a stock solution of 20 mM, which was then kept at −20°C.

### Histology

After being treated with 4% paraformaldehyde, the tissue slices were embedded in paraffin sections. The slides were then dewaxed and stained with hematoxylin and eosin (H&E). Kidney sections were sequentially stained with Gill’s hematoxylin, differentiated with 0.3% acid alcohol, and incubated with eosin and phloxine. Finally, the sections were dehydrated and mounted.

### Real-Time PCR

The TRIzol RNA reagent (Invitrogen, Carlsbad, CA) was used to extract RNA from kidney tissue and cells; the concentration was determined using NanoDropTM (Ultraspec 2,000; Pharmacia Biotech, Cambridge, United Kingdom). The Smart Cycler II System was used to make cDNA (Cepheid, Sunnyvale, CA). SYBR green was employed as a dye to identify DNA in real-time PCR. The relative amounts of mRNA were measured by a Rotor-GeneTM 3000 Detector System and real-time PCR (Corbette Research, Mortlake, New South Wales, Australia). The primer sequences are provided in Supplementary Table S1.

### Immunohistochemical Staining

Kidney specimens were preserved in 10% formalin and submerged in phosphate buffer saline (PBS). The tissues were paraffin-embedded and sliced into 3 μm pieces. Following deparaffinization and rehydration, the standard immunohistochemistry (IHC) technique was performed as previously described. Primary anti-F4/80 antibody (MCA497GA, Bio-Rad, Hercules, CA) and horseradish peroxidase-conjugated anti-mouse IgG secondary antibody (Dako, Glostrup, Denmark) were used for immunohistochemical staining. Visual fields were chosen randomly from digital pictures of each slide under ×20 magnification. ImageJ software was used for quantitative analysis of the stained sections.

### Cell Viability Analysis

NRK52E cells were grown on 96-well plates and starved with no FBS medium before being treated with the specified dosage of H_2_O_2_, MA, or vehicle alone (DMSO). The viability of cells was determined using an EZ-Cytox1000 kit (Dogen, Daejeon, South Korea) following the manufacturer’s instructions. At 450 nm, the absorbance of cells was analyzed using a microplate reader (Bio-Tek Instruments, Winooski, VT, United States).

### Western Blotting

RIPA buffer was used to lyse total proteins from kidneys or cells that had been frozen in liquid nitrogen (Thermo Scientific, Waltham, MA, United States). Tissue/cell debris was removed after brief centrifugation at 13,000 x g, and the supernatant was collected. Total protein was determined using a BCA Protein Assay kit and its manufacturer’s guidelines (Thermo Scientific, Waltham, MA, United States). The following steps of western blot analysis were performed as described previously.

### Nuclear Extract Preparation

Cells were lysed according to the manufacturer’s instructions using the NE-PER nuclear extraction reagent (NER; Pierce Biotechnology, Rockford, IL, United States). NRK52E cells were collected and centrifuged at 13,000 x g for 2 min. After draining the supernatant, the dried cell pellets were treated with ice-cold cytoplasmic extraction reagent I (CER I), cold CER II, and NER. Finally, the nuclear extract fraction was collected and protein concentrations were measured by the BCA assay.

### TUNEL Staining

According to the manufacturer's instructions, the ApopTag Plus peroxidase *In Situ* Apoptosis Detection Kit (Chemicon International; Temecula, CA, United States) and the DeadEndTM Fluorometric TUNEL System (Promega Corporation) were used to detect apoptosis *in vivo* and *in vitro*, respectively. The slices of kidneys and cells were examined using light and electron microscopy (at ×40 magnification).

### Mitochondria and Cytoplasm Isolation

A mitochondria isolation kit (Thermo Scientific, Waltham, MA, United States) was used to examine the subcellular localization of Bax and Bcl-2 in NRK52E cells. Mitochondria and cytoplasm fractions were isolated according to the manufacturer’s instructions.

### Statistical Analysis

One-way ANOVA was used to perform Tukey’s post hoc test for parametric data and the Kruskal–Wallis test with Dunn’s multiple comparisons for nonparametric tubular damage data. For continuous variables, parametric variables were expressed as mean ± SD, and nonparametric variables were expressed as median and interquartile (25th and 75th percentile) ranges, *p* values <0.05 were considered statistically significant. For all statistical studies, GraphPad Prism 9 was used for all statistical analyses (GraphPad Software, San Diego, CA).

## Result

### Maslinic Acid Protects Renal Function and Ameliorates Renal Histopathological Damages in Ischemia/Reperfusion Injury Model

**FIGURE 1:**
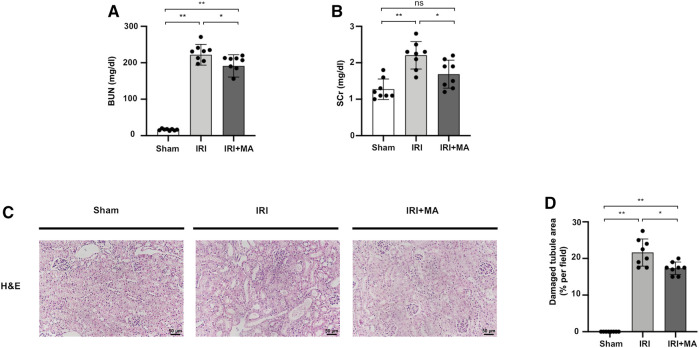
MA protects renal function and ameliorates renal histopathological damages in IRI model. **(A,B)** BUN and SCr in sham, IRI, and IRI + MA group after 72 h after bilateral I/R surgery. **(C)** Histological changes were assessed by H&E staining. Original magnification = ×20, Bar = 50 μm. **(D)** Kruskal–Wallis test for tubular damage, n = 8. *p < 0.05, **p < 0.01, ns: no significance. BUN, blood urea nitrogen; SCr, serum creatinine; HE, hematoxylin and eosin; MA, maslinic acid; IRI, ischemia-reperfusion injury.

As shown in Figure 1, blood urea nitrogen (BUN) and serum creatinine (SCr) were increased in the IRI group compared with those of the sham group, and were obviously improved in MA treated mice (Figures 1A,B). The H&E stain showed the histopathological changes of the IRI kidney include massive infiltration of interstitial inflammatory cells, accumulation of cell debris, and tubular dilation in the IRI group. However, MA treatment limited these changes to a certain extent (Figures 1C,D).

### Maslinic Acid Attenuates Renal Macrophage Infiltration and Inflammation in the IRI Model

**FIGURE 2:**
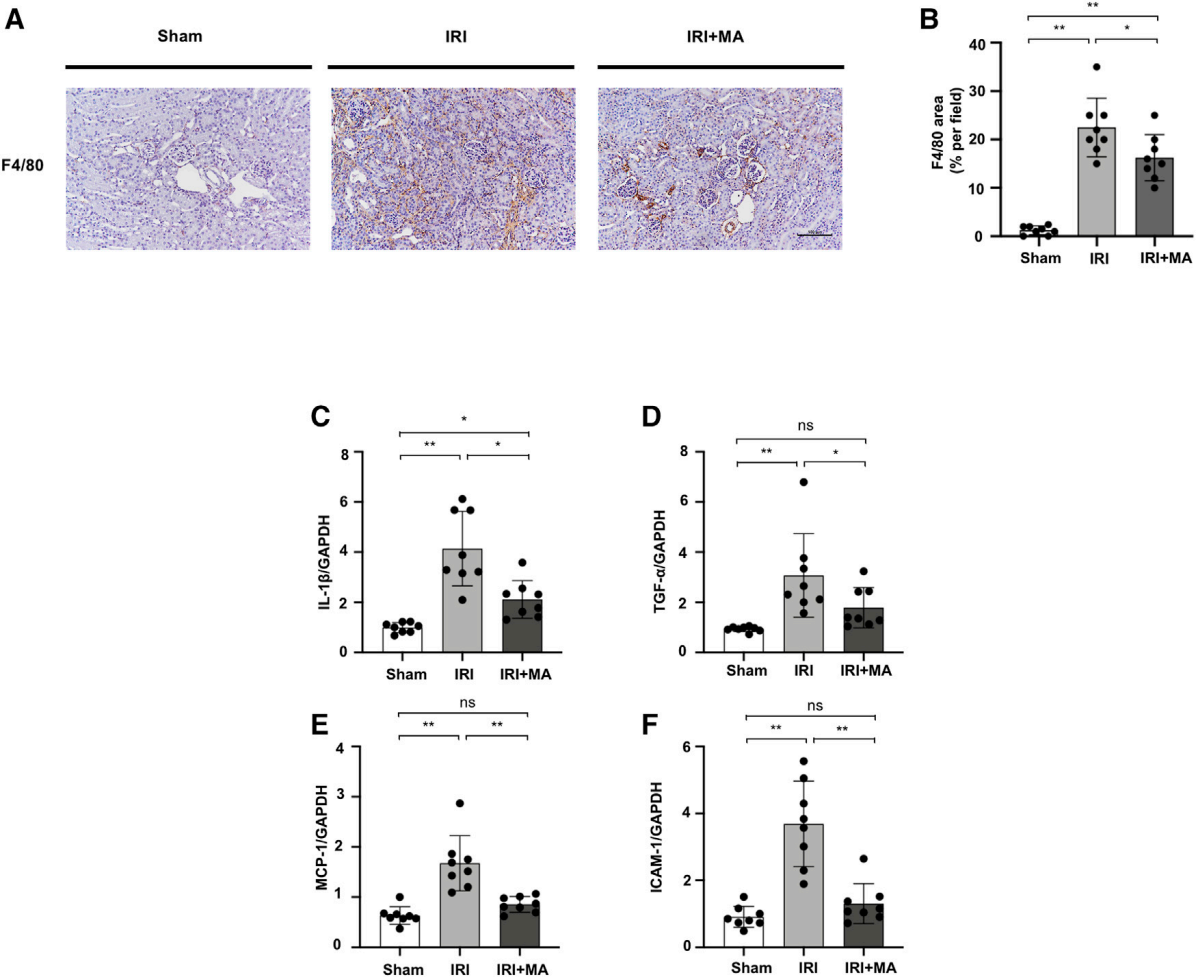
MA attenuates renal macrophage infiltration and inflammation in IRI model. **(A)** Immunohistochemistry was used to identify F4/80 expression in kidneys. Original magnification = ×20. Bar = 100 μm. **(B)** The data were shown as mean ± SD, n = 8. *p < 0.05, **p < 0.01, ns: no significance. **(C–F)** RT-PCR was used to assess the mRNA expression levels of IL-1β, TNF-α, MCP-1 and ICAM-1 in sham, IRI, and IRI + MA group (24 h pre-treatment MA) after 72 h after bilateral I/R surgery. GAPDH expression was used to standardize the results. MA, maslinic acid; IRI, ischemia reperfusion injury.

After IRI, F4/80, the macrophage biomarker, was positive in IRI kidneys, demonstrating the interstitial infiltration of inflammatory cells, which was significantly decreased by MA treatment (Figures 2A,B). Real-time PCR revealed that mRNA levels of IL-1β, TNF-α, MCP-1, and ICAM-1 were increased in IRI kidneys, which was counteracted by MA treatment (Figures 2C–F).

### Maslinic Acid Protects NRK52E Cells From H_2_O_2_ Induced Inflammation

**FIGURE 3:**
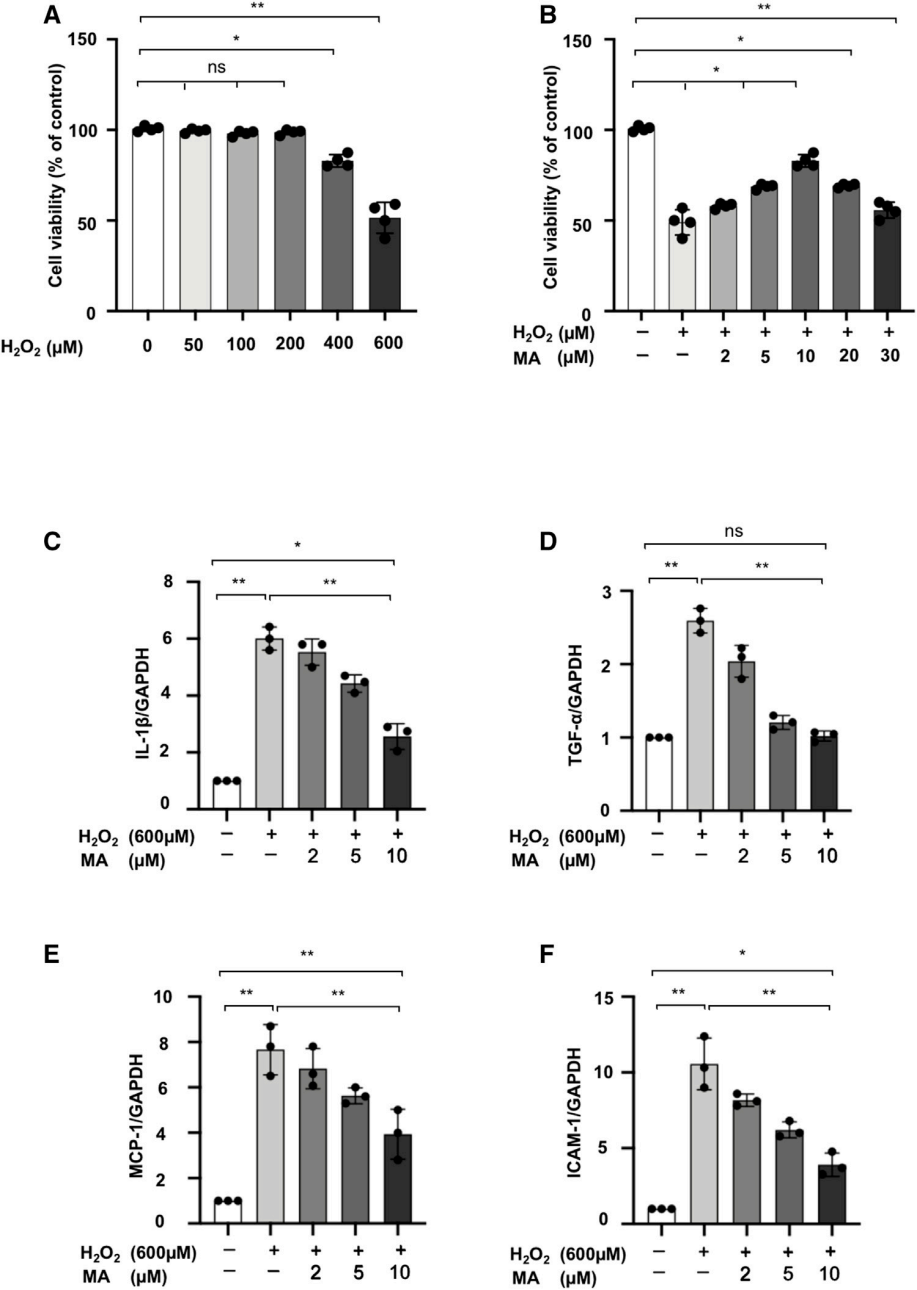
MA protects NRK52E cells from H_2_O_2_ induced inflammation. **(A)** NRK52E cells were exposed to H_2_O_2_ (0–600 μM) for 6 h and cytotoxicity assays were conducted. **(B)** NRK52E cells were pre-treated with different doses of MA for 2 h before being incubated with or without 600 μM H_2_O_2_ for 6 h. Cell viability was all examined by MTT assay. **(C–F)** RT-PCR was used to detect the mRNA expression levels of IL-1β, TNF-α, MCP-1 and ICAM-1 in H_2_O_2_-treated NRK52E cells. After starving with no FBS medium for 24 h, cells were treated with MA for 2 h before H_2_O_2_ (600 μM) for 1 h. GAPDH expression was used to standardize the results. The data were shown as the mean ± SD, n = 3. *p < 0.05, **p < 0.01, ns: no significance. MA, maslinic acid; RT-PCR, Real-Time PCR.

We assessed the cytotoxicity of MA and H_2_O_2_ on the NRK52E cell line. At concentrations ranging from 0 to 600 μM for 6 h, we finally determined that the dose of 600 μM of H_2_O_2_ reduced cell viability by 50% (Figure 3A). Next, we chose the 600 μM of H_2_O_2_ with different concentrations of MA for 2 h pre-treatment for another 6 h. Decreased cell viability induced by H_2_O_2_ was recovered by MA at a concentration of 2, 5, 10 μM. Therefore, 2, 5, and 10 μM MA were selected in our following experiments (Figure 3B). We then evaluated the effect of MA on H_2_O_2_-induced inflammation. In H_2_O_2_-stimulated NRK52E cells, mRNA levels of IL-1β, TNF-α, MCP-1, and ICAM-1 increased; however, the changes were partially reversed by MA pre-treatment in a dose-dependent manner (Figures 3C–F).

### Maslinic Acid Downregulated NF-κB Signaling and Inhibited P65 Nuclear Translocation in H_2_O_2_ Treated NRK52E Cells

**FIGURE 4:**
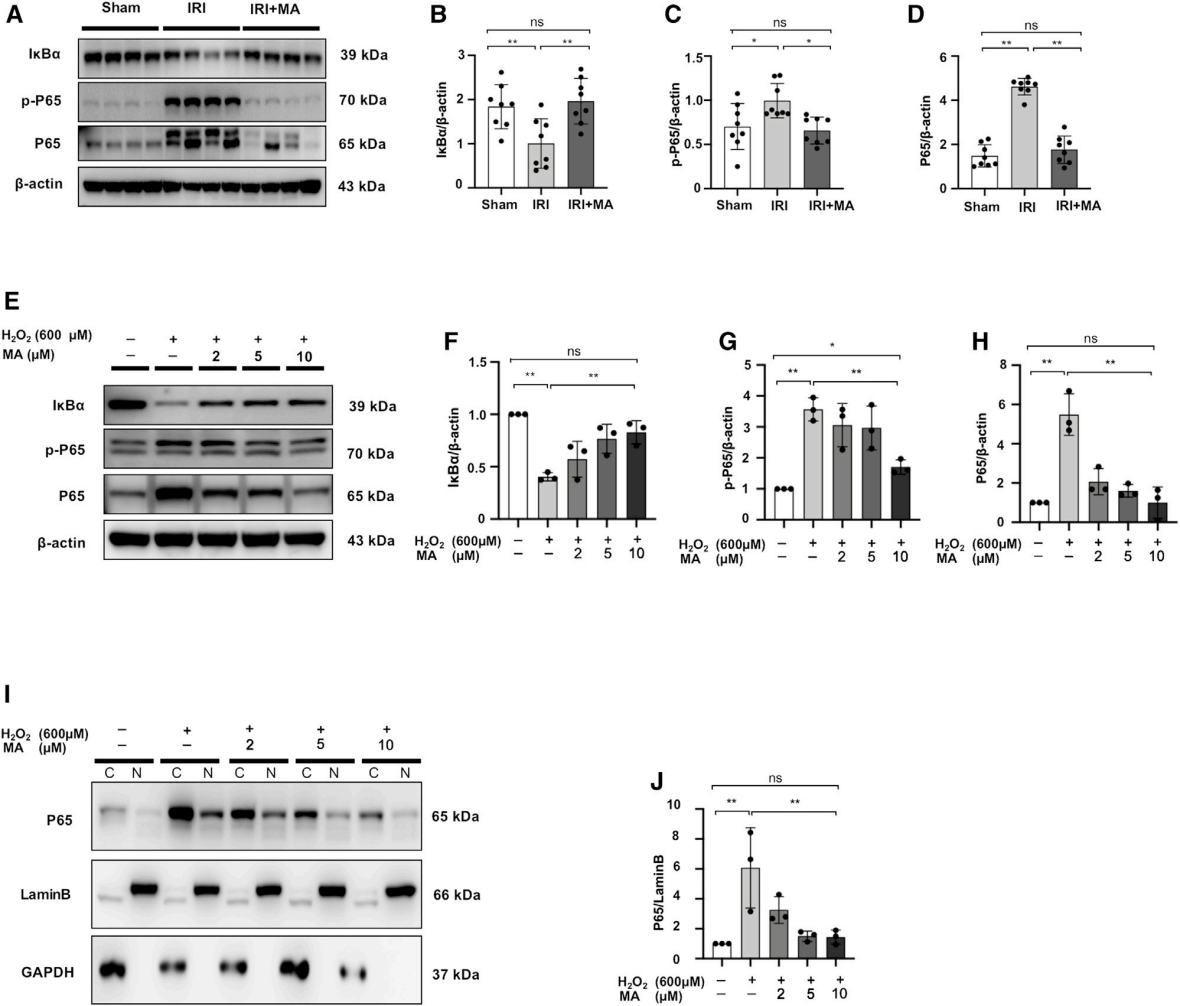
MA downregulated NF-κB signaling and inhibited P65 nuclear translocation in H_2_O_2_-treated NRK52E cells. **(A)** Western blot was used to identify and quantify IκBα, p-P65, P65 in the kidney. **(B–D)** The data significance was expressed as mean ± SD, n = 8. *p < 0.05, **p < 0.01, ns: no significance. **(E)** Western blot detection and quantification of IκBα, p-P65, and P65 expression were shown in H_2_O_2_-treated NRK52E cells. After starving with no FBS medium for 24 h, cells were treated with MA for 2 h following with H_2_O_2_ (600 M) for 1 h **(F–H)** The data were expressed as mean ± SD, n = 3. *p < 0.05, **p < 0.01, ns: no significance. **(I)** Western blotting analysis showed P65 nuclear translocation in H_2_O_2_-stimulated NRK52E cells with or without MA, CER/NER buffer was used to separate cytoplasmic and nuclear proteins. The cytoplasmic and nuclear fractions were standardized by Lamin B and GAPDH, respectively. After starving with no FBS medium for 24 h, cells were pre-treated with MA for 2 h before stimulated with H_2_O_2_ (600 M) for 1 h. **(J)** The data were expressed as mean ± SD, n = 3. *p < 0.05, **p < 0.01, ns: no significance. MA, maslinic acid; C, Cytoplasmic protein; N, nuclear protein.

The expression of p-P65, P65 expression was upregulated in the kidneys 72 h after IRI, as well as decreased in the expression of IκBα protein. MA treatment inhibited the increased expression of p-P65 and P65 and recovered the IκBα expression (Figures 4A–D). The protein expression of p-P65 and P65 in H_2_O_2_-treated NRK52E cells was higher than those in the control group, which were reduced by MA pretreatment. Meanwhile, the H_2_O_2_-induced loss of IκBα expression gradually rebounded after MA administration in a dose-dependent condition (Figures 4E–H). We further determined whether MA could regulate the nuclear translocation of NF-κB in NRK52E cells treated with H_2_O_2_. MA dose-dependently attenuates nuclear translocation from the cytosol of P65 (Figures 4I,J). The result further confirmed the negatively regulated function of MA on NF-κB signaling.

### Maslinic Acid Suppresses Renal Apoptosis in IRI Mice Models

**FIGURE 5:**
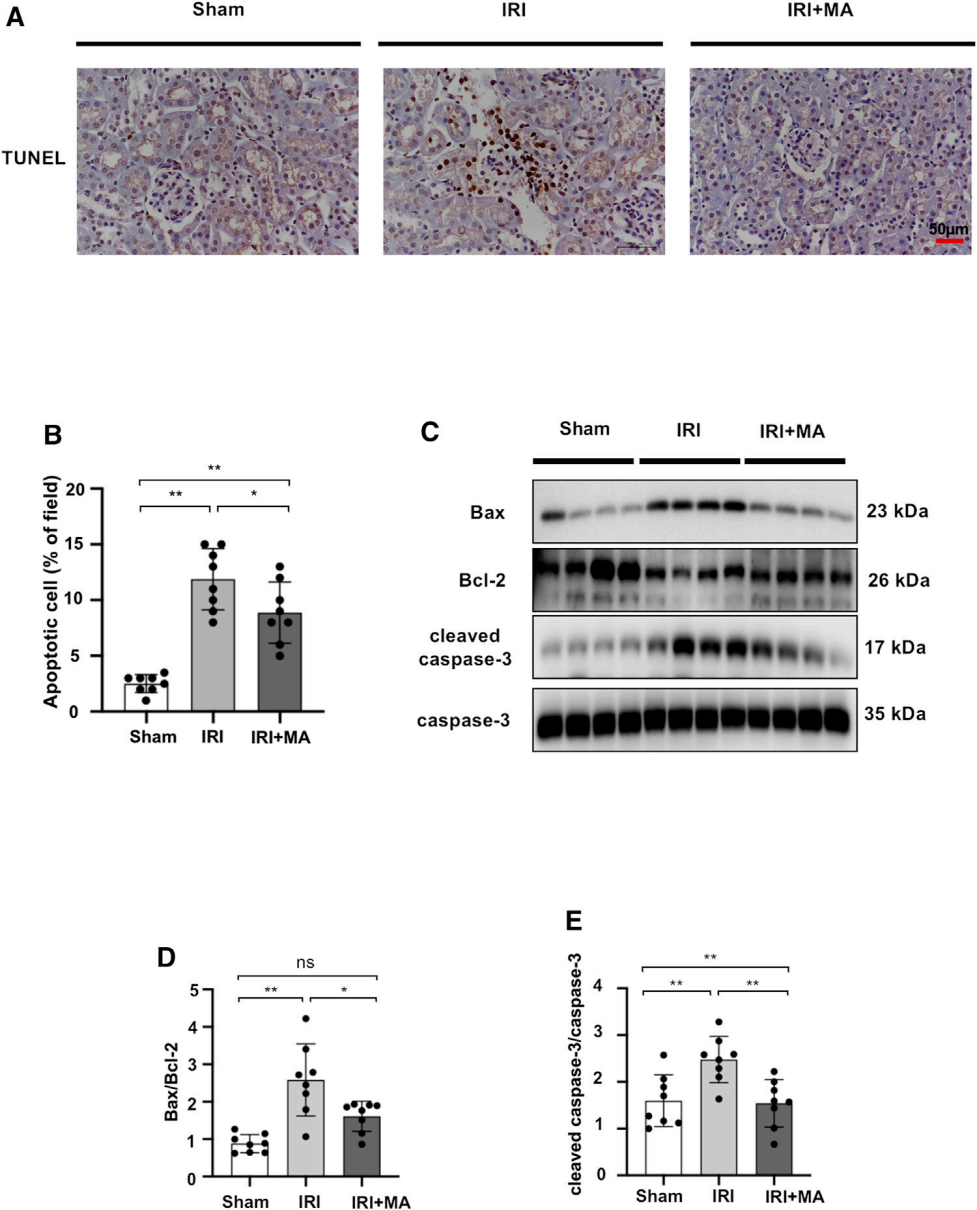
MA suppresses renal apoptosis in IRI mice model. **(A)** TUNEL assay performed on sections of kidneys after IRI treated or not with MA. Original magnification = ×20, Bar = 50 μm. **(C)** Western blots were used to quantify Bax, Bcl-2, cleaved caspase-3, and caspas-3 protein levels in sham, IRI, and IRI + MA group after 72 h after bilateral I/R surgery. **(B,D,E)** The data were expressed as mean ± SD, n = 8. *p < 0.05, **p < 0.01, ns: no significance. MA, maslinic acid; IRI, ischemia reperfusion injury.

To assess whether MA treatment affects the extent of apoptosis in IRI kidneys, we stained apoptotic cells in kidney tissues with the TUNEL assay. As shown in the images, the number of apoptotic cells increased in mice after IRI surgery, and most of the apoptotic cells were located in the renal tubular region. We found that the MA treatment group had fewer TUNEL-positive cells compared to the IRI group (Figures 5A,B). In addition, the Bax/Bcl-2 ratio and cleaved caspase-3/caspase-3 ratio exhibited increasing expression in the IRI group, while a significant down-regulation of these markers was observed in the MA treatment group (Figures 5C–E). These results indicate that MA could attenuate renal apoptosis following IRI in mice.

### Maslinic Acid Hinders H_2_O_2_-Stimulated Apoptosis of NRK52E Cells

**FIGURE 6:**
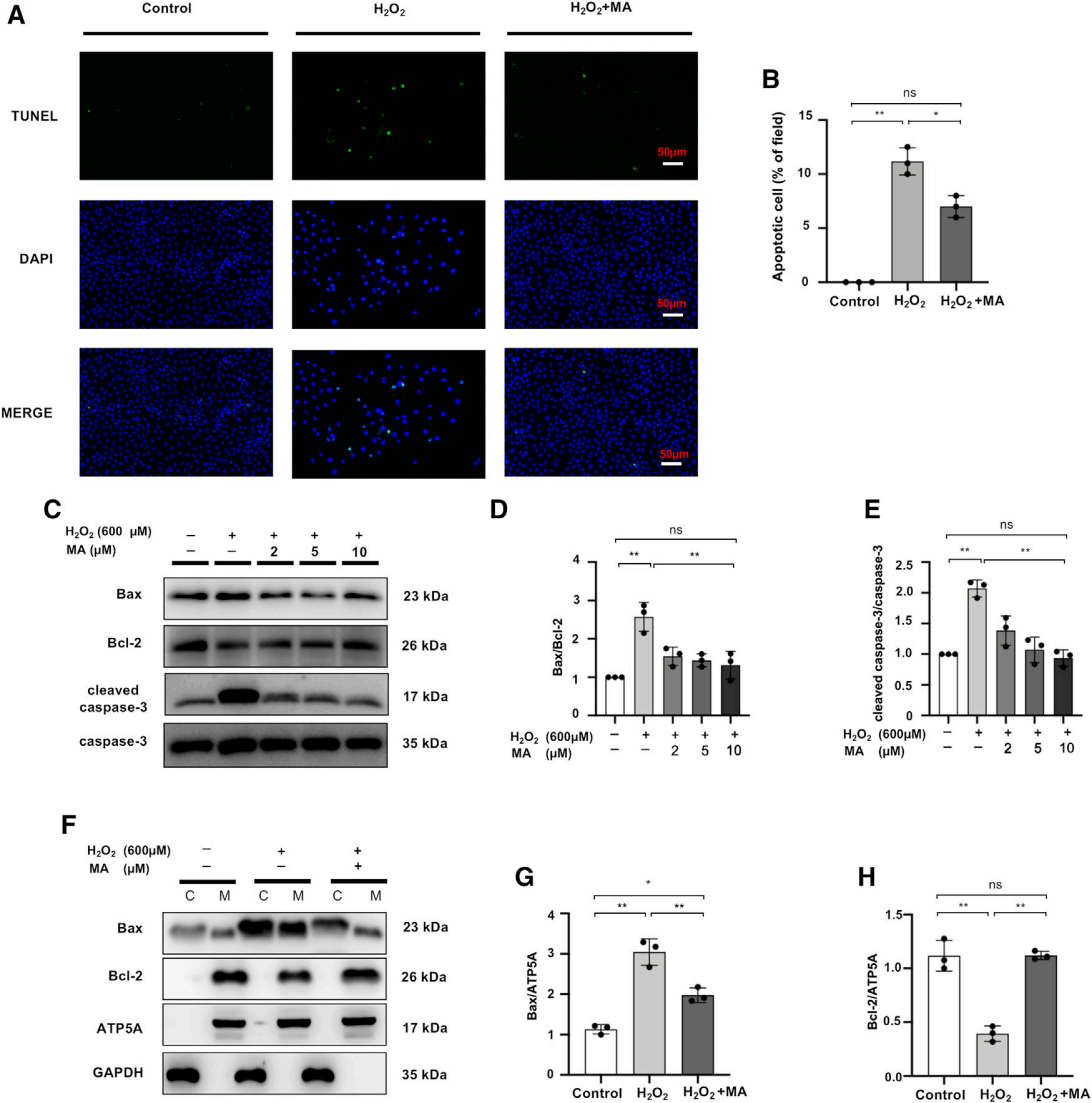
MA hinders H_2_O_2_-stimulated apoptosis of NRK52E cells. **(A)** TUNEL assay performed on sections of H_2_O_2_ induced NRK52E cells pre-treated or not with MA. Original magnification = ×40, Bar = 50 μm. **(C)** Western blots were used to detect Bax, Bcl-2, cleaved caspase-3, and caspase-3. After starving with no FBS medium for 24 h, cells were pre-treated with MA for 2 h before stimulated with H_2_O_2_ (600 M) for 6 h. **(F)** Bax, Bcl-2 mitochondrial and cytoplasmic fragments were detected by Western blots; the mitochondrial and cytoplasmic fractions were standardized by ATP5A and β-actin, respectively. **(B,D,E,G,H)** The data were expressed as mean ± SD, n = 3. *p < 0.05, **p < 0.01, ns: no significance. MA, maslinic acid.

After 6 h of exposure to 600 μM H_2_O_2_, the results of TUNEL staining to detect the apoptosis of NRK52E cells showed that MA treatment significantly decreased apoptotic cell numbers in H_2_O_2_ treated NRK52 cells (Figures 6A,B). Also, compared to the control group, the levels of Bax and cleaved caspase-3 in NRK52E cells exposed to 600 μM H_2_O_2_ for 6 h were remarkably higher than those of the control group, whereas the protein expression level of Bcl-2 was lower in the H_2_O_2_ group than in the other two groups. MA treatment reduced the Bax/Bcl-2 ratio and decreased cleaved caspase-3/caspase-3 levels (Figures 6C–E). We further evaluated the expression of Bax and Bcl-2 in mitochondrial and cytoplasmic fragments. The results showed that Bax was partially transferred from the cytoplasm to the mitochondria in H_2_O_2_-stimulated NRK52E cells, while MA inhibited cytoplasmic to mitochondrial translocation. Bcl-2 expression was mainly in mitochondria and was inhibited by H_2_O_2_ induction, with a rebound in expression after MA treatment (Figures 6F–H).

### Maslinic Acid Inhibits Activation of MAPK Signaling *in vivo* and *in vitro*

**FIGURE 7:**
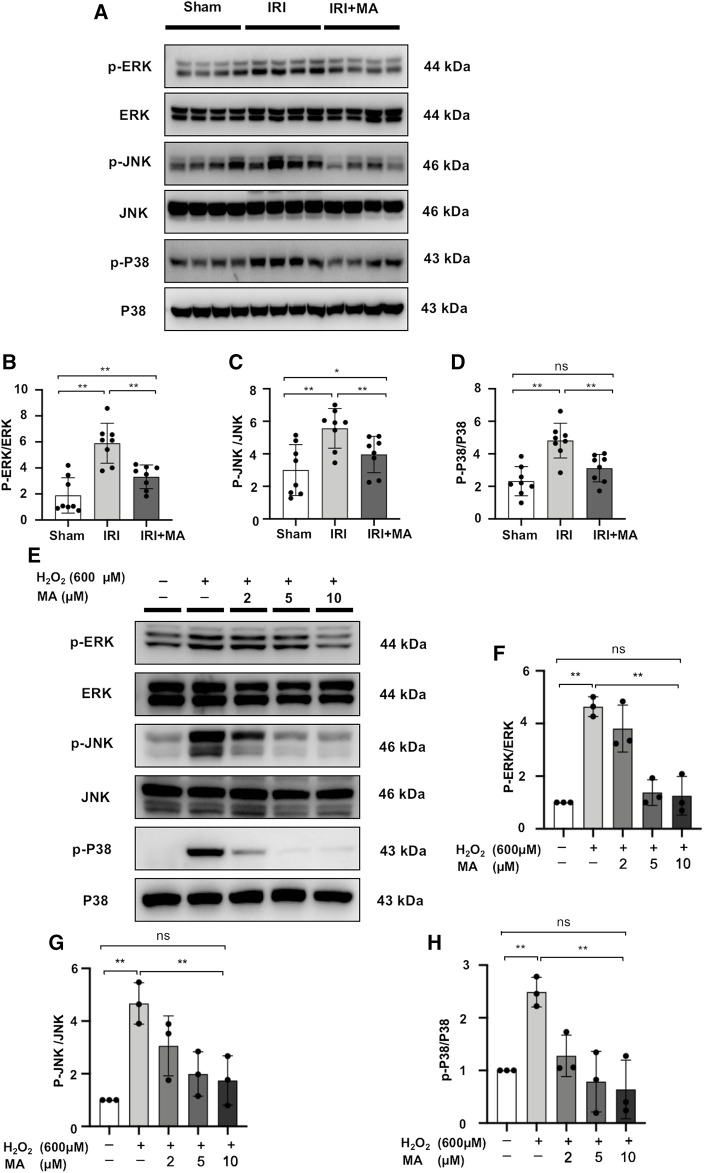
MA inhibits activation of MAPK signaling *in vivo* and *in vitro*. **(A)** Western blot was used to identify and determine the protein levels of p-ERK1/2, ERK1/2, p-JNK, JNK, p-P38, and P38 in sham, IRI, and IRI + MA group (24 h pre-treatment MA). **(B–D)** The data were expressed as mean ± SD, n = 8. *p < 0.05, **p < 0.01, ns: no significance. MA, maslinic acid; IRI, ischemia reperfusion injury. **(E)** Western blot detection and analysis of p-ERK1/2, ERK1/2, p-JNK, JNK, p-P38, and P38 expression in H_2_O_2_-treated NRK52E cells. After starving with no FBS medium for 24 h, cells were pre-treated with MA for 2 h before being treated with H_2_O_2_ (600 M) for 1 h **(F–H)** The data were expressed as mean ± SD, n = 3. *p < 0.05, **p < 0.01, ns: no significance. MA, maslinic acid.

We quantified the main components of the MAPK signaling pathway in kidney tissue and H_2_O_2_-stimulated NRK52E cells by Western blotting. The expression levels of ERK1/2, JNK and P38 were similar in each group *in vivo* and vitro. However, the protein expression of p-ERK1/2, p-JNK and p-P38 was up-regulated in IRI mice and H_2_O_2_ induced NRK52E cells, which was counteracted by MA treatment (Figures 7A–H). These results indicate that MAPK activation during renal IRI could be inhibited by MA.

### Maslinic Acid Upregulates Nuclear Factor Erythroid 2-Related Factor 2 Protein Accumulation in Ischemia/Reperfusion Injury Mice and H_2_O_2_-Stimulated NRK52E Cells

**FIGURE 8:**
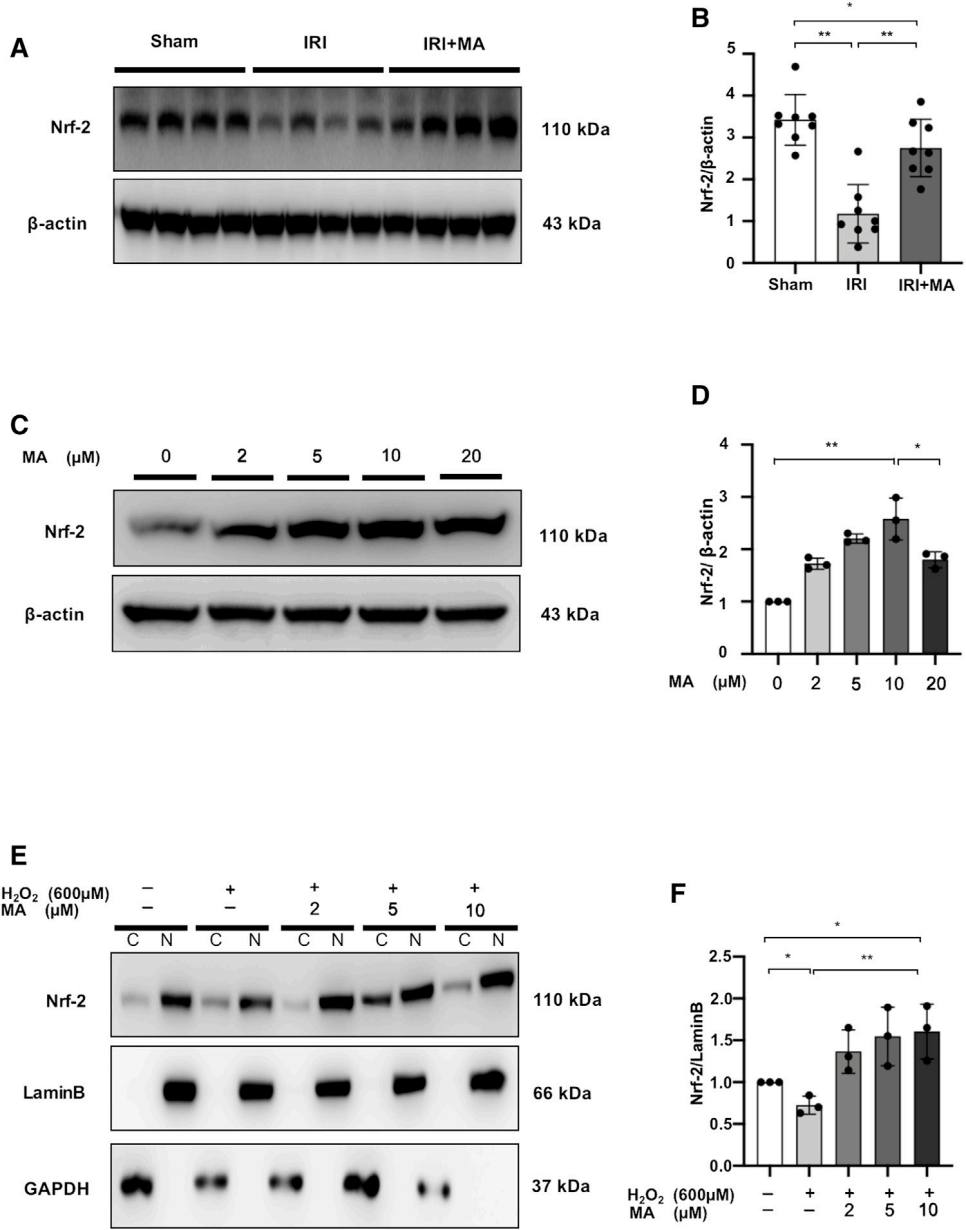
MA upregulates nuclear factor erythroid 2-related factor 2 (Nrf-2) protein accumulation in IRI mice and H_2_O_2_-stimulated NRK52E cells. **(A)** Western blot was used to identify and determine the protein levels of Nrf-2 in sham, IRI, and IRI + MA group (24 h pre-treatment MA). **(B)** The data were expressed as mean ± SD, n = 8. *p < 0.05, **p < 0.01, ns: no significance. MA, maslinic acid; IRI, ischemia reperfusion injury. **(C)** Western blot detection and analysis of Nrf-2 expression in MA-treated NRK52E cells. After starving with no FBS medium for 24 h, cells were treated with MA for 2 h and havast. **(E)** MA reduced Nrf-2 nuclear accumulation in H_2_O_2_-stimulated NRK52E cells. CER/NER buffer was used to separate cytoplasmic and nuclear proteins. GAPDH and Lamin B used to indicate the cytoplasmic and nuclear fractions, respectively. After starving with no FBS medium for 24 h, cells were pretreated with MA for 2 h before being treated with H_2_O_2_ (600 M) for 1 h **(D,F)** The data were expressed as mean ± SD, n = 3. *p < 0.05, **p < 0.01, ns: no significance. MA, maslinic acid; C stands for cytoplasmic protein; and N stands for nuclear protein.

Nrf-2 expression was reduced in the kidneys of IRI mice, and treatment with MA partially reversed the declining trend (Figures 8A,B).

In NRK52E cells, we examined the possibility of Nrf-2 induction by MA. Treatment of different doses (0–10 μM) of MA for 2 h showed increased expression of Nrf-2 in a dose-dependent manner in NRK52E cells, while the Nrf-2 expression decreased under 20 μM MA treatment (Figures 8C, D). Next, we determined the ability of MA to regulate the nuclear accumulation of Nrf-2 in NRK52E cells treated with H_2_O_2_. We found that MA dose-dependently (within 10 μM) increased the intranuclear expression of Nrf-2 (Figures 8E,F). These results suggest the positive regulatory function of MA on the Nrf-2 signaling factor.

### NF-κB Inhibitor Regulates the Nuclear Localization of P65 and Nrf-2 and Downregulates Inflammatory Markers Level in H_2_O_2_-Stimulated NRK52E Cells

**FIGURE 9:**
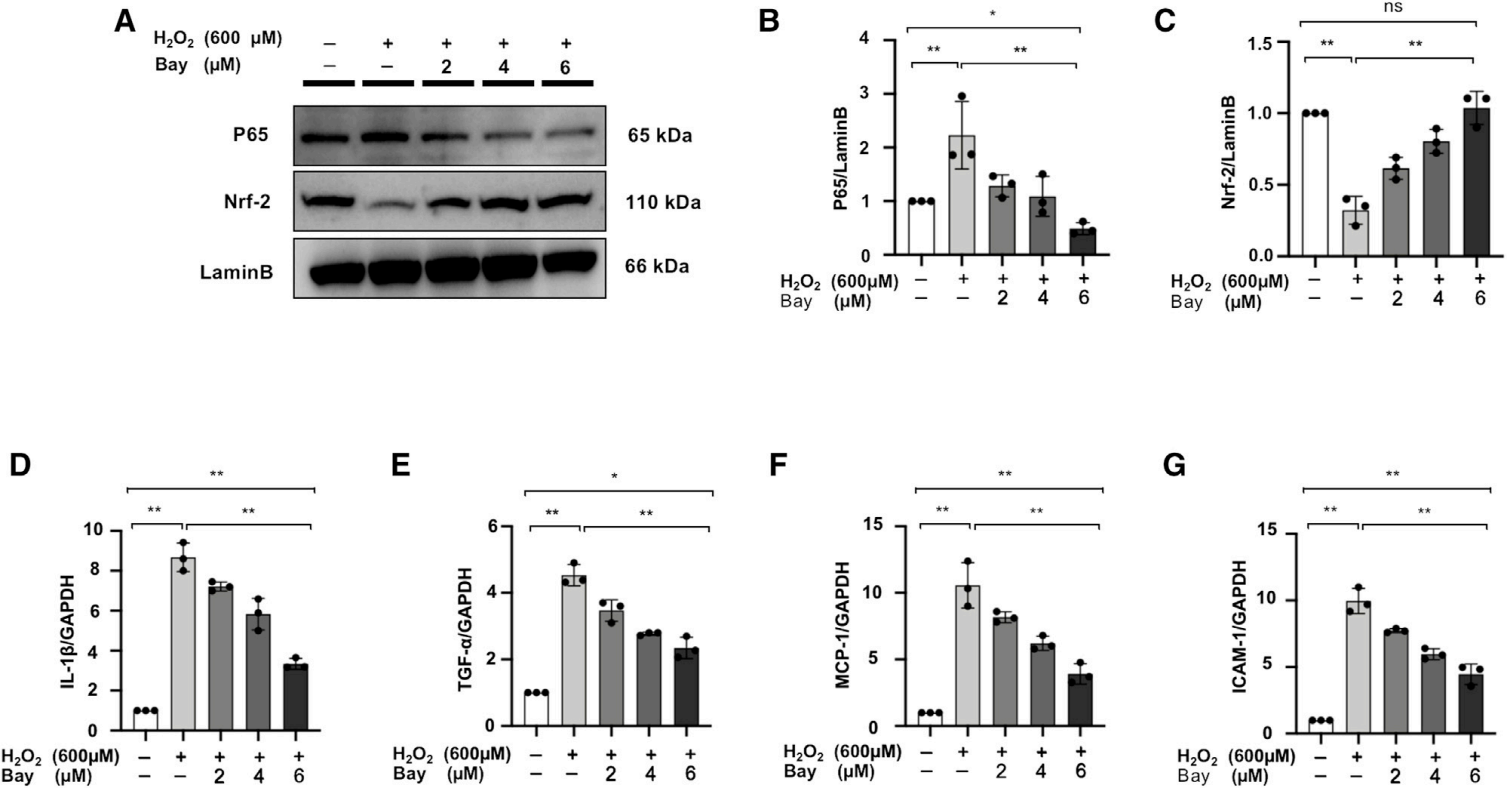
NF-κB inhibitor regulates the nuclear localization of P65 and Nrf-2 and downregulates inflammatory markers level in H_2_O_2_-stimulated NRK52E cells. **(A)** In H_2_O_2_-stimulated NRK52E cells, Bay treatment reduced or enhanced nuclear level of P65 and Nrf-2. NER buffer was used to extract nuclear protein. **(B–C)** The data were expressed as mean ± SD, n = 3. *p < 0.05, **p < 0.01, ns: no significance. **(D–G)** RT-PCR was used to detect the mRNA expression levels of IL-1β, TNF-α, MCP-1 and ICAM-1 in H_2_O_2_-treated NRK52E cells. After starving with no FBS medium for 24 h, cells were pre-treated with Bay for 2 h before being treated with H_2_O_2_ (600 M) for 1 h. GAPDH expression was used to standardize the results. The data were expressed as mean ± SD, n = 3. *p < 0.05, **p < 0.01, ns: no significance. RT-PCR, real-time PCR.

We further demonstrated that the NF-κB inhibitor, bay treatment, dose-dependently decreased P65 nuclear expression and increased Nrf-2 expression in H_2_O_2_-stimulated NRK52E cells (Figures 9A–C). Meanwhile, mRNA expression levels of IL-1β, TNF-α, MCP-1 and ICAM-1 were upregulated in H_2_O_2_-stimulated NRK52E cells, which was attenuated by Bay treatment in a dose-dependent manner (Figures 9D–G).

### MAPK Signaling Inhibitors SP/PD/SB Decrease Apoptotic Protein Expression in H_2_O_2_-Stimulated NRK52E Cells

**FIGURE 10:**
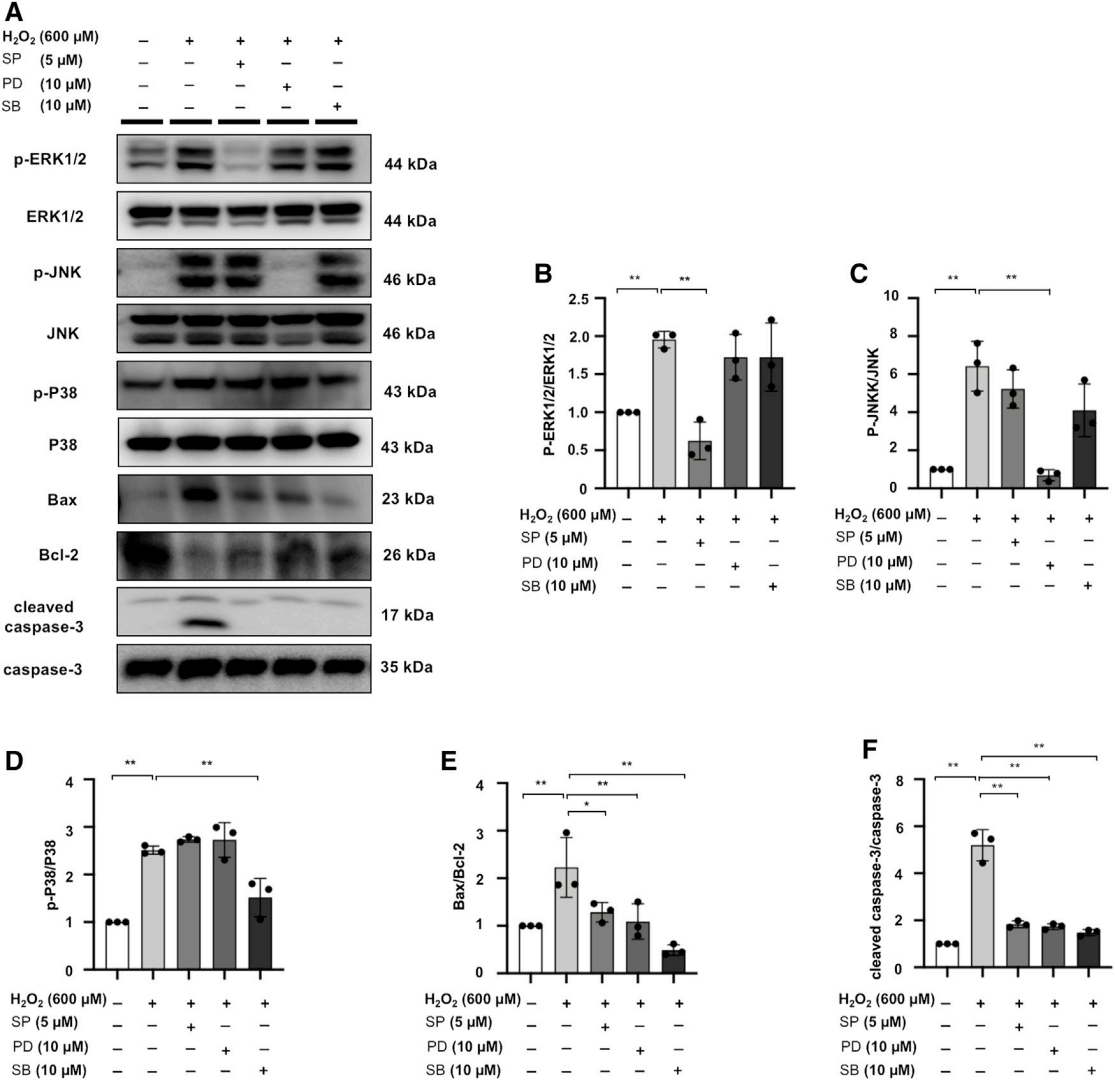
MAPK signaling inhibitors SP/PD/SB decrease apoptotic protein expression in H_2_O_2_-stimulated NRK52E cells. **(A)** The expression of p-ERK1/2, ERK1/2, p-JNK, JNK, p-P38, P38, Bax, Bcl2, and cleaved caspase3 in H_2_O_2_-treated NRK52E cells was identified by western blot and quantified by densitometry. Cells were pretreated with SP/PD/SB for 2 h following starvation with no FBS media, and then treated with H_2_O_2_ (600 M) for 1 h or 6 h harvest. **(B–F)** The data were expressed as mean ± SD, n = 3. *p < 0.05, **p < 0.01, ns: no significance.

H_2_O_2_ treatment increased Bax/Bcl-2 and cleaved caspase-3/caspase-3 ratio in NRK52E cells, while the expression of these markers was counteracted by SP, PD, SB treatment in H_2_O_2_-stimulated NRK52E cells (Figures 10A–F).

### Maslinic Acid Pre-treatment 1 h IRI Mice with No Significant Improvement in Renal Function, Inflammation, and Apoptosis Markers Compared to Ischemia/Reperfusion Injury Mice (Protocol 2)

We examined the differences in BUN and SCr in each group. There was an increase in the IRI group compared to the sham group, while there was no obvious improvement in the 1 h pre-treatment MA group (Supplementary Figure S1A,B). Next, we investigated the effect of 1 h pre-treatment MA on the mRNA expression of pro-inflammatory molecules in IRI-damaged kidneys. The mRNA levels of IL-1β, TNF-α, MCP-1 and ICAM-1 were significantly increased in the IRI group compared to the sham group. These upregulated pro-inflammatory cytokines or chemokines were not significantly improved after the administration of MA (Supplementary Figure S1C–F). Moreover, the ratio level of Bax/Bcl-2 and cleaved caspase-3/caspase-3 was increased in the IRI group, and one dose of MA 1 h before IRI surgery did not reverse these ratio levels of apoptotic markers (Supplementary Figure S1G–I).

## Discussion

Renal IRI is a common cause of AKI and can ultimately lead to irreparable kidney damage as well as excessive mortality from AKI (Gueler et al., 2004; Lameire et al., 2013). Previous studies have confirmed that therapies such as anti-inflammatory and anti-apoptotic treatments after renal IRI might be advantageous (Lutz et al., 2010; Havasi and Borkan, 2011; Gao et al., 2016). In this investigation, we found the renal protective effects of MA in the IRI model, including counteracting-inflammatory and apoptotic properties by suppressing NF-κB and MAPK signaling, at least in part.

The efficacy and safety of MA have been proven in previous studies. Sánchez-González et al. reported that daily oral administration of 50 mg/kg of MA for 28 days did not induce any signs of toxicity during the experimental period (Sánchez-González et al., 2013). During the treatment period, body weight did not change before and after treatment, and hematological and biochemical variables were not affected by treatment. Histopathological examination of organs showed no differences between control and treated mice. Also, Mokhtari et al. found that MA did not produce any cytotoxic effect, even at the highest doses used (Mokhtari et al., 2020). Also, in our previous study, we treated MA at 20 mg/kg for 7 days and observed no side effects on mice (Sun et al., 2021).

The inflammatory cascade is a key player engaged in the pathophysiology of renal IRI, inducing kidney tissue destruction by releasing a variety of mediators (Kinsey et al., 2008; Bonventre and Yang, 2011). MA exerts anti-inflammatory effects on lung tissue by regulating iNOS through inhibition of NF-κB and p-STAT-1 (Lee et al., 2020). In the present study, the morphological changes evaluated by H&E staining showed that MA significantly reduced inflammatory infiltration, renal tubular cell necrosis, and cast formation. In addition, MA treatment reduced the interstitial infiltration of F4/80 and this phenomenon is consistent with the previous studies (Liou et al., 2019). Ischemia-reperfusion leads to immune activation and massive expression of adhesion molecules (e.g., ICAM-1) that infiltrate into the interstitium to enhance adhesion between inflammatory cells and endothelial cells (Rabb et al., 1995). Kelly et al. reported that the gene-deficient adhesion molecule ICAM-1 could protect against renal injury in an animal model of renal ischemia (Kelly et al., 1996). In addition, renal tubular epithelial cells can also contribute to the inflammatory response to renal IR injury by producing various pro-inflammatory cytokines (e.g., TNF-α, IL-1β) and chemokines (e.g., MCP-1) (Haq et al., 1998; Sung et al., 2002; Spurgeon et al., 2005). In the present study, pro-inflammatory cytokines and cell adhesion molecules were decreased *in vitro* and *in vivo* following MA administration, indicating that MA may ameliorate IRI by inhibiting the inflammatory response. There is evidence that NF-κB activation occurs efficiently in numerous organs in response to ischemia and hypoxia, including the kidney, brain, liver, and myocardium. Donnahoo et al. revealed that in the early stages of renal ischemia, with or without reperfusion, NF-κB is activated and TNF-α bioactivity is increased in the kidney (Donnahoo et al., 2000). Some evidence suggests that NF-κB activation in the renal tubular epithelium after ischemia exacerbates tubular injury and exacerbates the adverse inflammatory response to ischemic AKI (Markó et al., 2016). In addition, MA has been shown to block the activity of the downstream NF-κB signaling pathway by inhibiting the degradation and phosphorylation of IκBα, thereby reducing nuclear localization, phosphorylation, and gene expression of P65 (Li et al., 2010; Huang et al., 2011). Therefore, it is reasonable to assume that the above reduction of proinflammatory cytokines is at least partially mediated by NF-κB related signaling pathway inhibition. The results showed that MA hindered the degradation of IκBα and reduced the high expression of P65 and p-P65 both *in vivo* and *in vitro* experiments, while being dose-dependent in blocking the entry of transcription factor P65 into the nucleus of NRK52E cells.

Most of the studies on the inter-relationship between MA and apoptosis have focused on the anticancer activity of MA, and over 100 µM treatment of MA on tumor cells has been proven to exert anti-proliferative and pro-apoptotic effects (Juan et al., 2008). Intriguingly, in the study of Ampofo et al., mitochondrial activity was found to be significantly reduced in endothelial cells treated with 20–40 µM of MA (Ampofo et al., 2019). Li et al. showed that MA effectively protected HAEC cells against apoptosis induced by high glucose within 0.25–1.0 μM (Li et al., 2017). Similarly, in the present study, TUNEL labeling revealed that the extent of renal tubular cell apoptosis was considerably enhanced in the IRI mouse model, as well as in H_2_O_2_-stimulated NRK52E cells. However, there is a significant reduction in the number of apoptotic cells in the relatively low dose administration of the MA group. Similarly, MA suppressed the Bax/Bcl-2 protein ratio and the protein expression of pro-apoptotic cleaved caspase-3 *in vivo* and *in vitro*. Meanwhile, our experimental results showed that H_2_O_2_ significantly accelerated the translocation of Bax from cytoplasm to mitochondria, and treatment with MA reversed the effects of H_2_O_2_ and inhibited the H_2_O_2_-induced translocation of Bax. Taken together, MA protects cells from oxidative stress by reversing the expression and translocation of Bax. Collectively, our study demonstrated that MA is a beneficial agent of anti-apoptosis in the renal IRI model, leading to hold apoptotic cell formation and regulate levels of pro-/anti-apoptotic proteins levels.

Mitogen-activated protein kinase (MAPK) is a family of structurally similar serine/threonine kinases that includes ERK1/2, JNK, and P38 (Cargnello and Roux, 2011). Although the intracellular events involved in apoptosis, necrosis, and post-traumatic survival have not been fully identified, the MAPK family is a recognized possible candidate for the aforementioned pathological processes (Wada and Penninger, 2004; Yue and López, 2020). Apoptosis and autophagy signal pathways in proximal tubular cells have been shown to involve extracellular signal-regulated ERK1/2, JNK, and P38 (Komoike et al., 2012). In ischemia-reperfused organs, ERK1/2, JNK and p38 tend to be activated immediately after reperfusion. and this activation is associated with tissue protection (Heidbreder et al., 2008; Kovalska et al., 2012). The present study observed that phosphorylation of ERK1/2, JNK and P38 was activated in IRI-operated mice and in H_2_O_2_-induced NRK52E cells; pre-treatment of MA reduced the phosphorylation of these MAPK family proteins. We previously proved that in p-cresyl sulfate-treated HK-2 cells, treated with a P38 inhibitor and a JNK inhibitor could reduce the expression of Bax and cleaved caspase-3, further effect apoptosis (Park et al., 2019). In addition, the present study proved that ERK1/2, JNK, and P38 inhibitors affect the expression of these apoptosis-related proteins. Taken together, MA may be another promising inhibitor of the MAPK signaling pathway and may play a role in slowing the apoptosis of cells induced by ischemia-reperfusion renal damage.

Interestingly, our findings suggest that the nuclear accumulation of Nrf-2 is enhanced by MA. Nrf-2 is known to regulate the expression of a range of cytoprotective genes and is also one of the transcription factors implicated in the control of inflammation (He et al., 2020). So far, many studies have shown a cross-talk between Nrf-2 and NF-κB pathways at the protein and transcriptional levels (Wardyn et al., 2015). In particular, it has been shown that NF-ĸB transcriptional activity and dependent gene transcription are significantly increased after Nrf-2 knockdown (Kim et al., 2010; Hwang et al., 2013). Another report proved that Nrf-2 inhibits NF-κB activation and protects against heart IRI (Fukunaga et al., 2020). Kumar et al. have proved that the effect of 2-week treatment with Bay could increase Nrf-2 protein levels in the sciatic nerve of diabetic rats (Kumar et al., 2012). Also, the present study proved that the application of Bay restored the expression of Nrf-2, which may be another mechanism of anti-inflammation by MA; the exact mechanism needs to be further investigated.

The therapeutic effects of MA on kidney damage were effective when they lasted for 3 days, from the day before IRI induction to 3 days after IRI. In our replicated trial, pre-treatment of one dose of MA before ischemic surgery couldn’t improve renal function, inflammation, and apoptosis. Therefore, the timing of administration and the cumulative dose are the determinants of the protective effect of MA.

In conclusion, MA treatment could reduce cellular inflammatory responses and apoptotic progression by modulating NF-κB and MAPK signaling pathways following ischemia-reperfusion stress. These findings suggest that MA holds promise as a potential candidate for the treatment of kidney disease in the future.

## Data Availability

The raw data supporting the conclusions of this article will be made available by the authors without undue reservation.
