# Supplementary material for: Maslinic Acid Attenuates Ischemia/Reperfusion-Induced Acute Kidney Injury by Suppressing Inflammation and Apoptosis Through Inhibiting NF-κB and MAPK Signaling Pathway
Source: Front Pharmacol. 2022 Apr 12;13:807452. doi: 10.3389/fphar.2022.807452 (PMC9039024; doi:10.3389/fphar.2022.807452)
Supplement: Supplementary file 1 [file Presentation1.PPTX]

## Slide 1
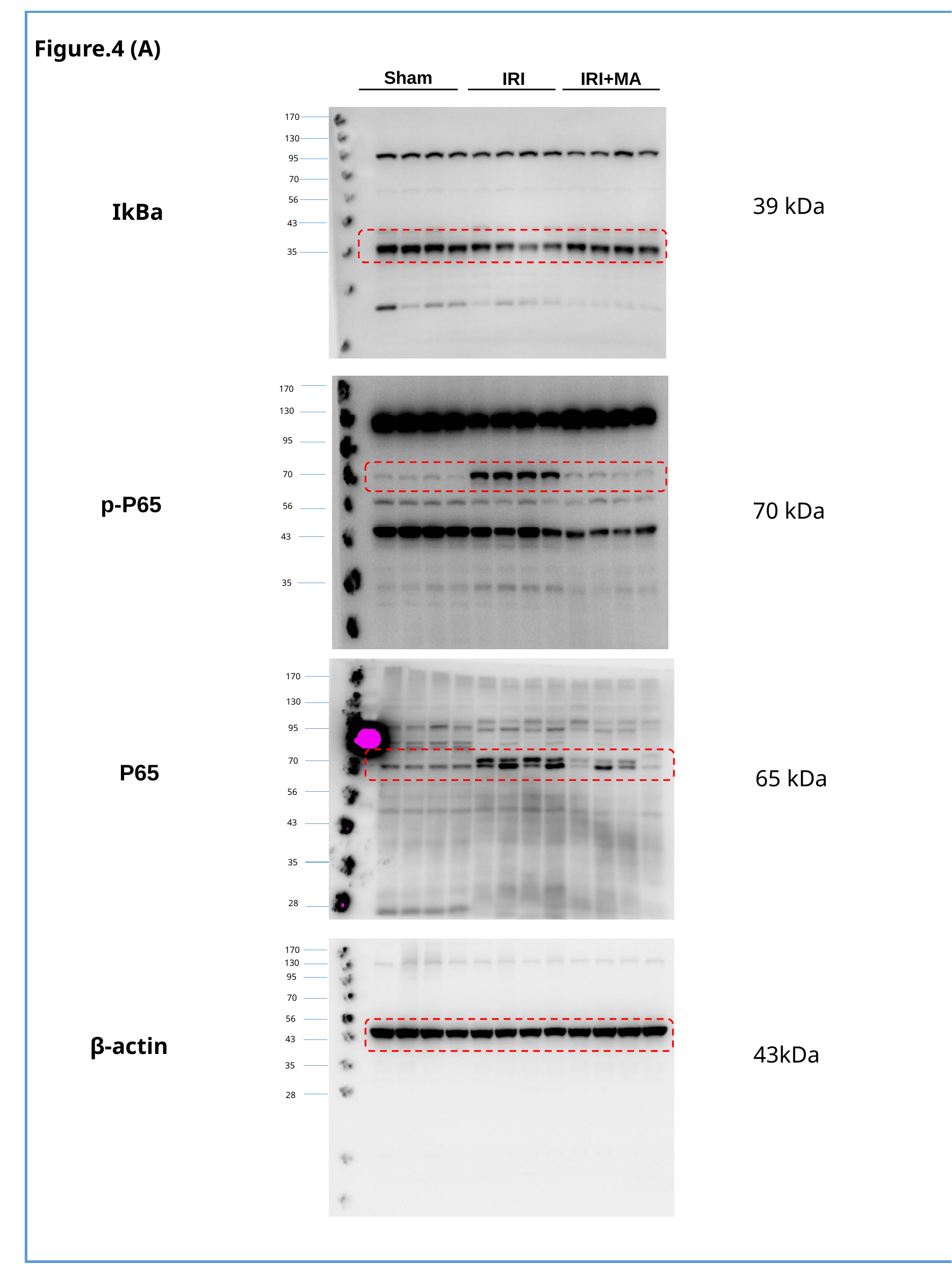

Figure.4 (A)
Sham
IRI
IRI+MA
170
130
95
70
 39 kDa
 70 kDa
 65 kDa
 43kDa
56
IkBa
p-P65
P65
43
35
170
130
95
70
56
43
35
170
130
 95
56
43
35
28
70
170
130
 95
56
43
35
28
70
β-actin
 70

## Slide 2
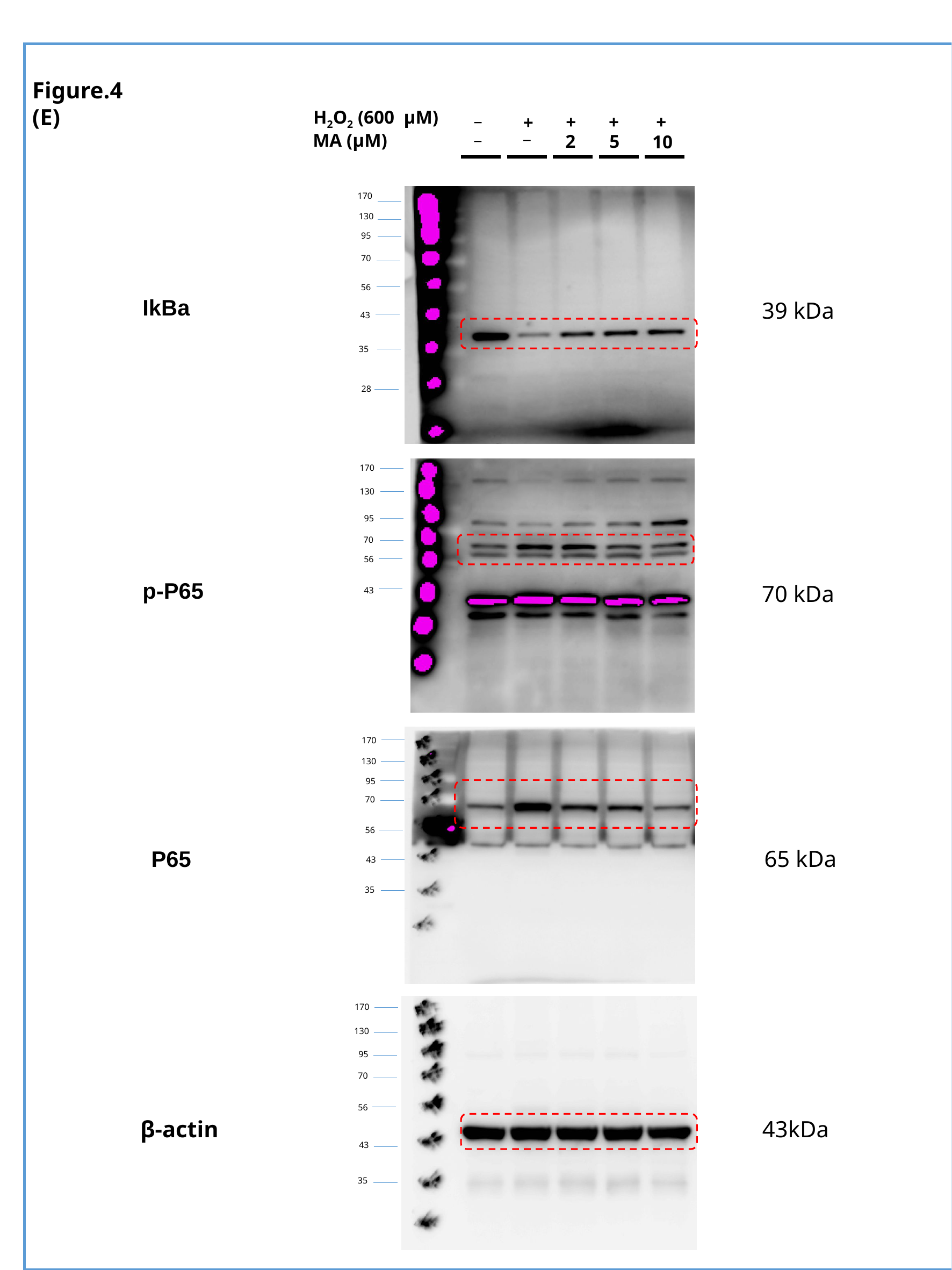

Figure.4 (E)
_
 H2O2 (600 µM)
+
+
 +
+
_
_
 MA (µM)
2
5
10
170
130
95
70
56
43
35
28
IkBa
 39 kDa
 70 kDa
 65 kDa
 43kDa
170
130
95
70
56
43
p-P65
170
130
95
70
56
43
35
P65
170
130
95
70
56
43
35
β-actin

## Slide 3
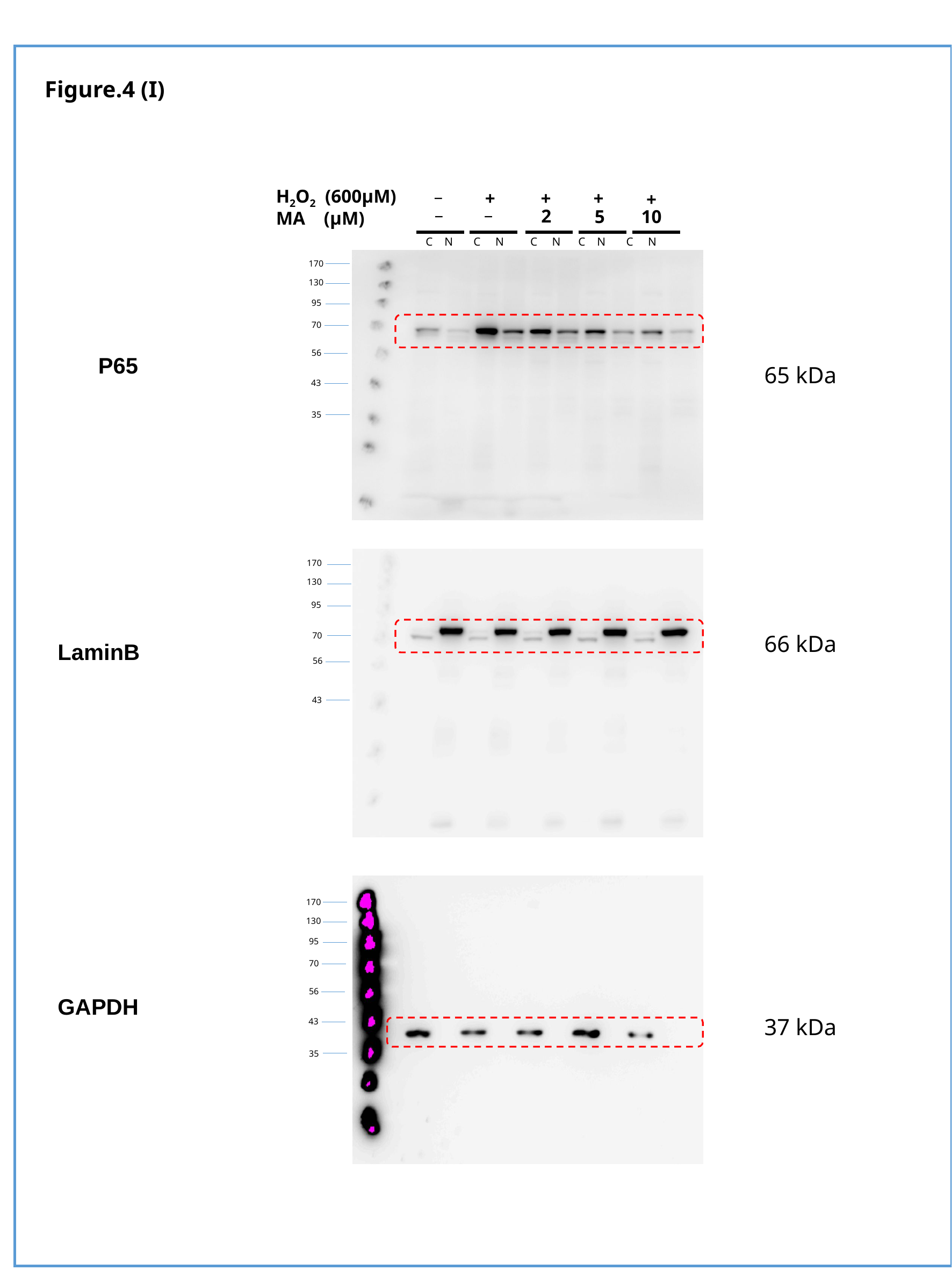

Figure.4 (I)
_
 H2O2 (600µM)
+
+
 +
 +
_
_
2
10
5
 MA (µM)
C N C N C N C N C N
170
130
95
70
56
43
35
P65
LaminB
GAPDH
 65 kDa
170
130
95
70
56
 66 kDa
43
170
130
95
70
56
43
35
 37 kDa

## Slide 4
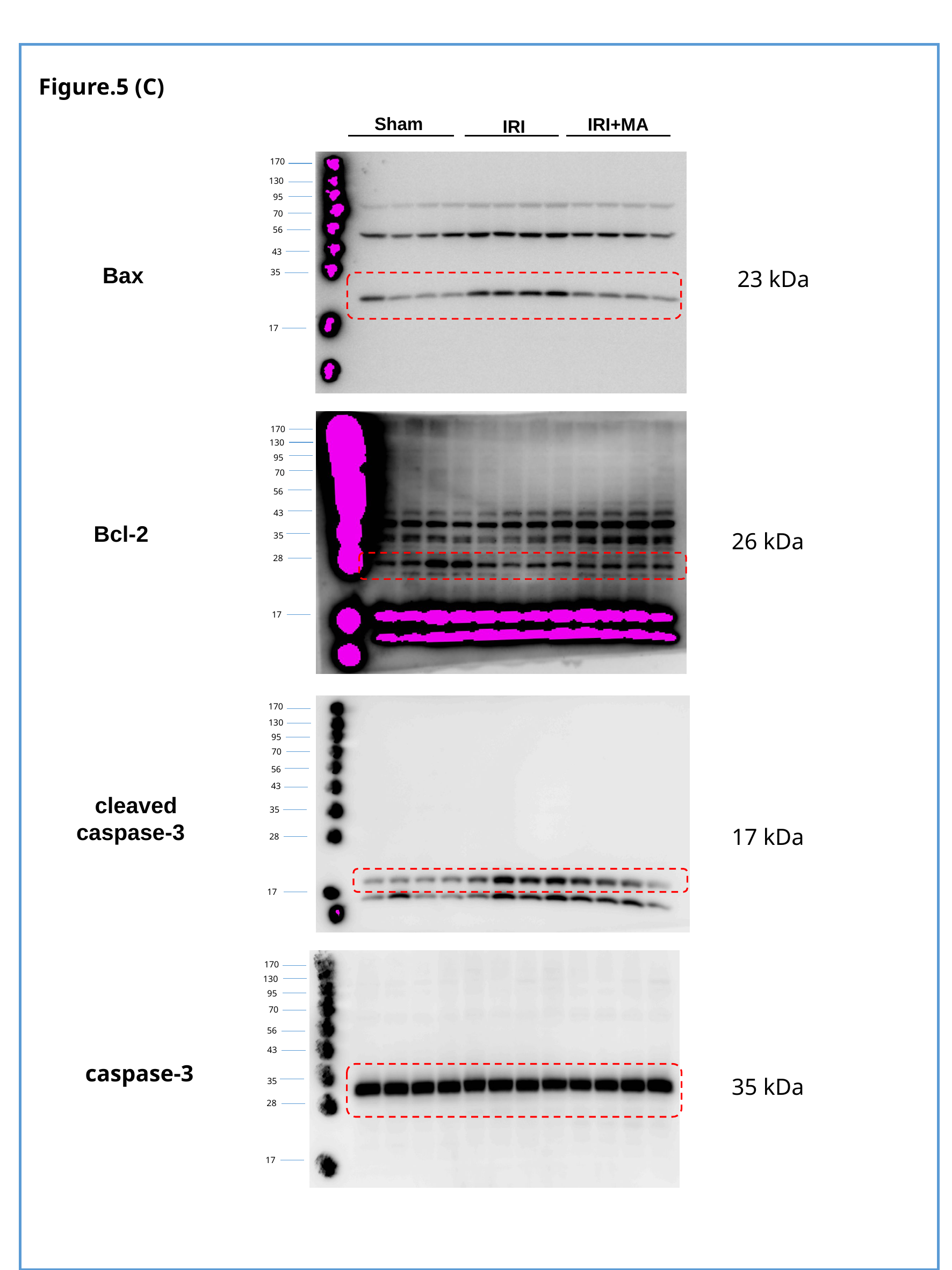

Figure.5 (C)
Sham
IRI+MA
IRI
170
130
95
70
56
43
35
17
Bax
Bcl-2
 23 kDa
 26 kDa
170
130
95
70
56
43
35
28
17
170
130
95
70
56
43
35
17
28
 cleaved caspase-3
caspase-3
 17 kDa
 35 kDa
170
130
95
70
56
43
35
28
17

## Slide 5
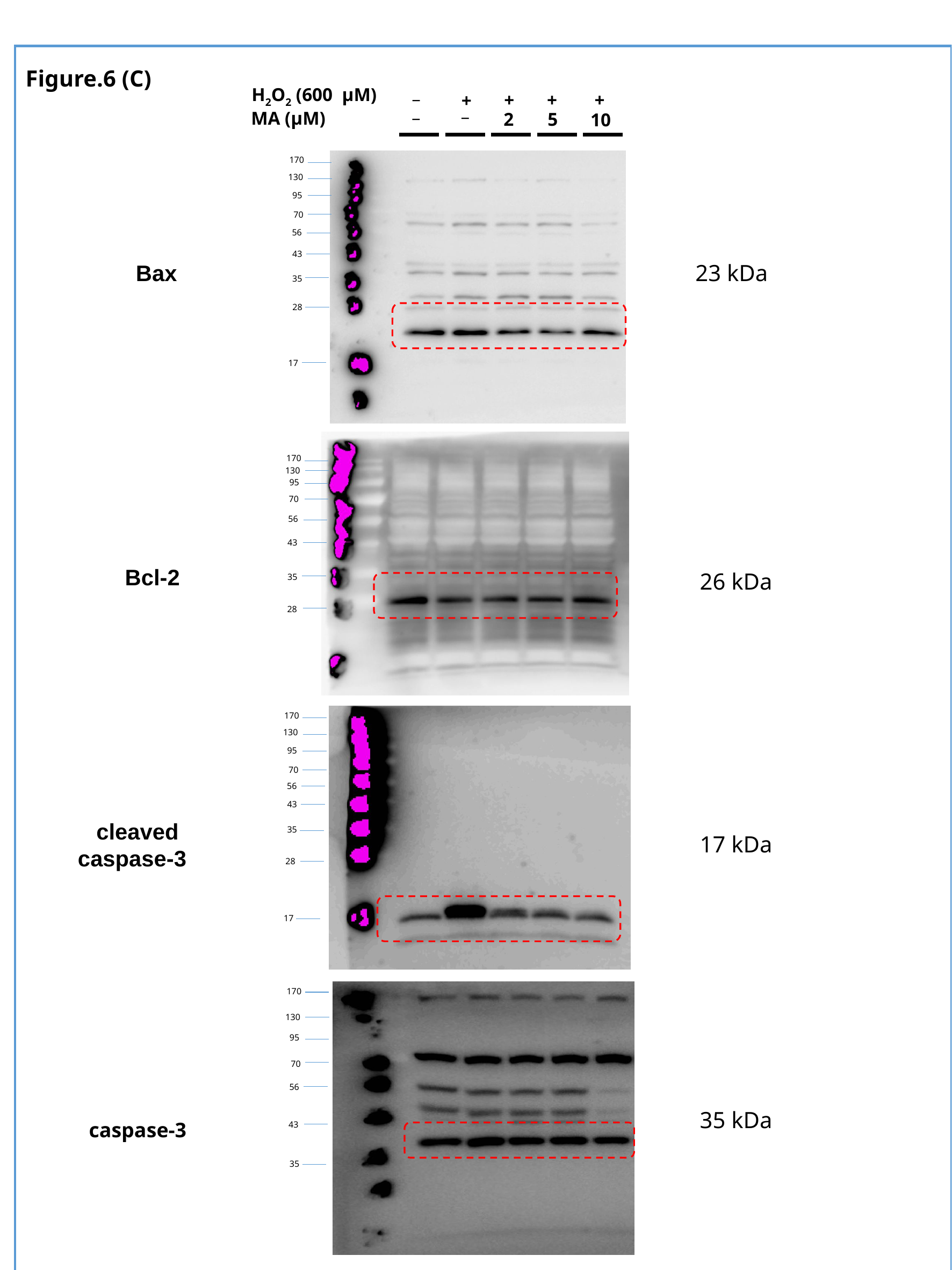

Figure.6 (C)
_
 H2O2 (600 µM)
+
+
 +
+
_
_
 MA (µM)
2
5
10
170
130
95
70
56
43
35
 23 kDa
 26 kDa
 17 kDa
 35 kDa
Bax
Bcl-2
 cleaved caspase-3
caspase-3
28
17
170
130
95
70
56
43
35
28
170
130
95
70
56
43
35
28
17
170
130
95
70
56
43
35

## Slide 6
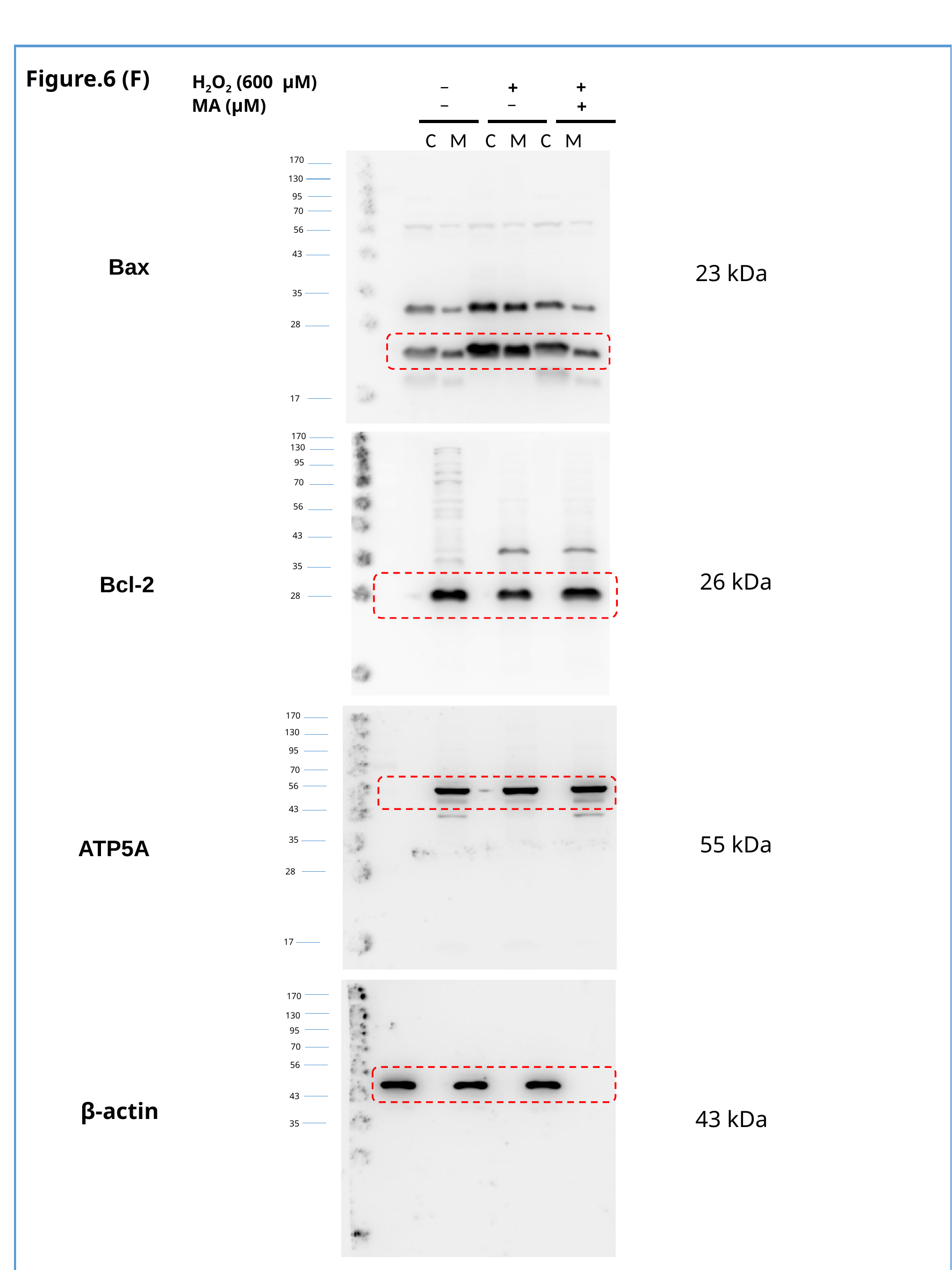

Figure.6 (F)
_
 H2O2 (600 µM)
+
+
_
_
 MA (µM)
+
C M C M C M
170
130
95
70
56
43
35
Bax
Bcl-2
 ATP5A
 23 kDa
 26 kDa
 55 kDa
 43 kDa
28
17
170
130
95
70
56
43
35
28
170
130
95
70
56
43
35
28
17
170
130
95
70
56
43
35
β-actin

## Slide 7
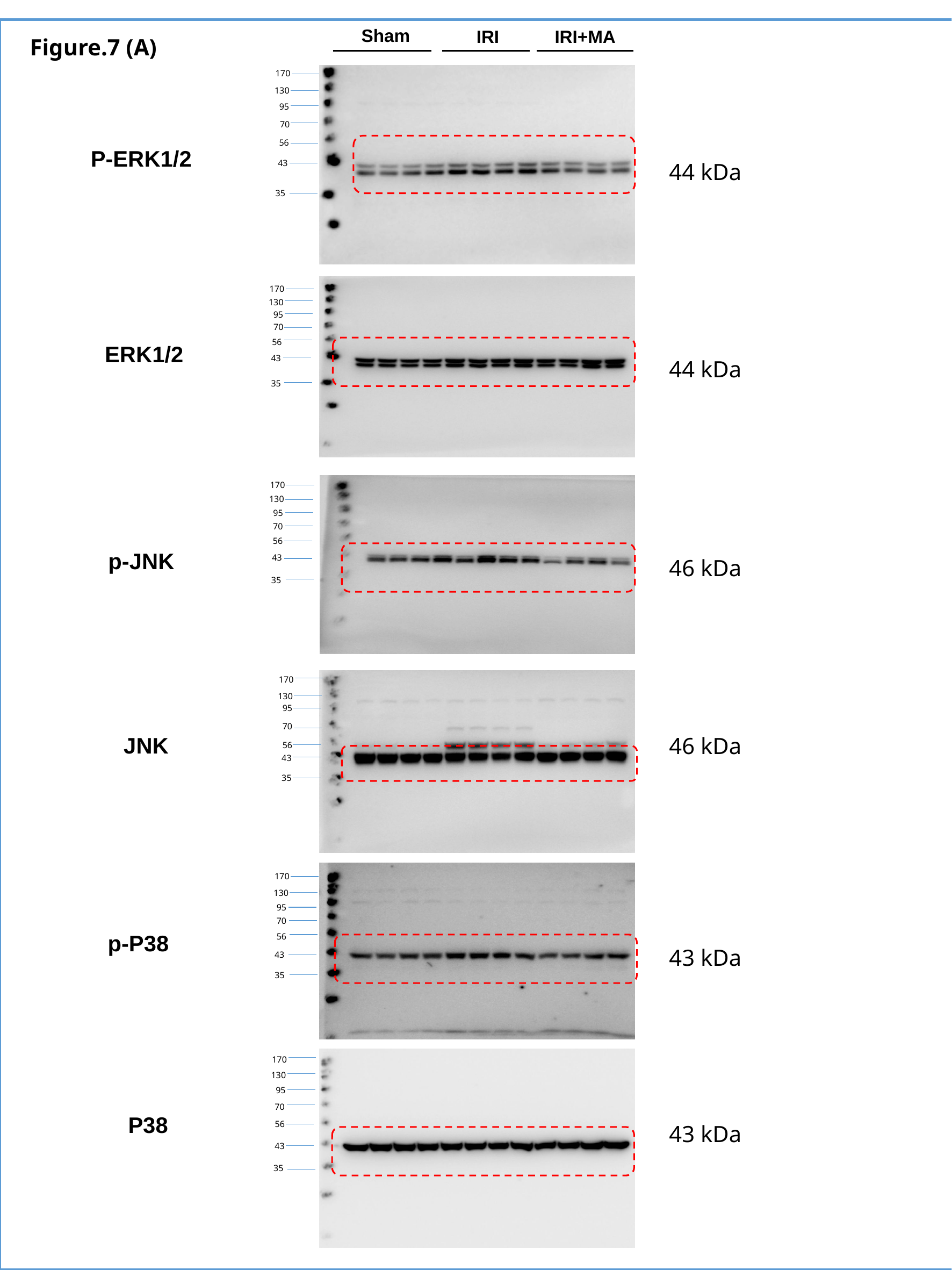

Sham
IRI
IRI+MA
Figure.7 (A)
170
130
95
70
56
43
35
P-ERK1/2
 ERK1/2
p-JNK
 JNK
p-P38
P38
 44 kDa
 44 kDa
 46 kDa
 46 kDa
 43 kDa
 43 kDa
170
130
95
70
56
43
35
170
130
95
70
56
43
35
170
130
95
70
56
43
35
170
130
95
70
56
43
35
170
130
95
70
56
43
35
 70

## Slide 8
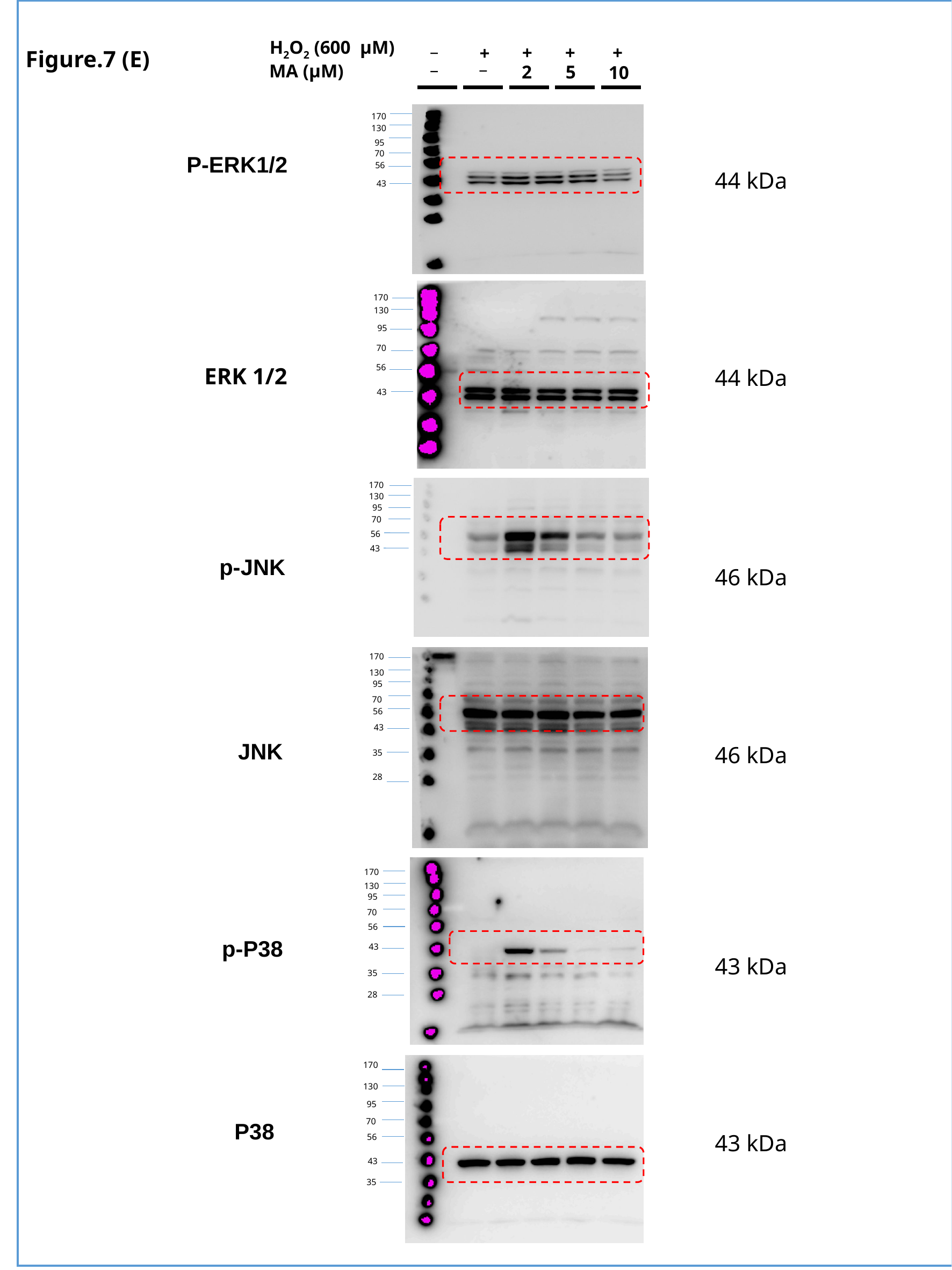

_
 H2O2 (600 µM)
+
+
 +
+
_
_
 MA (µM)
2
5
10
Figure.7 (E)
170
130
95
70
56
43
P-ERK1/2
 ERK 1/2
p-JNK
 JNK
p-P38
P38
 44 kDa
 44 kDa
 46 kDa
 46 kDa
 43 kDa
 43 kDa
170
130
95
70
56
43
170
130
95
70
56
43
170
130
95
70
 56
43
35
28
170
130
95
70
 56
43
35
28
170
130
95
70
 56
43
35

## Slide 9
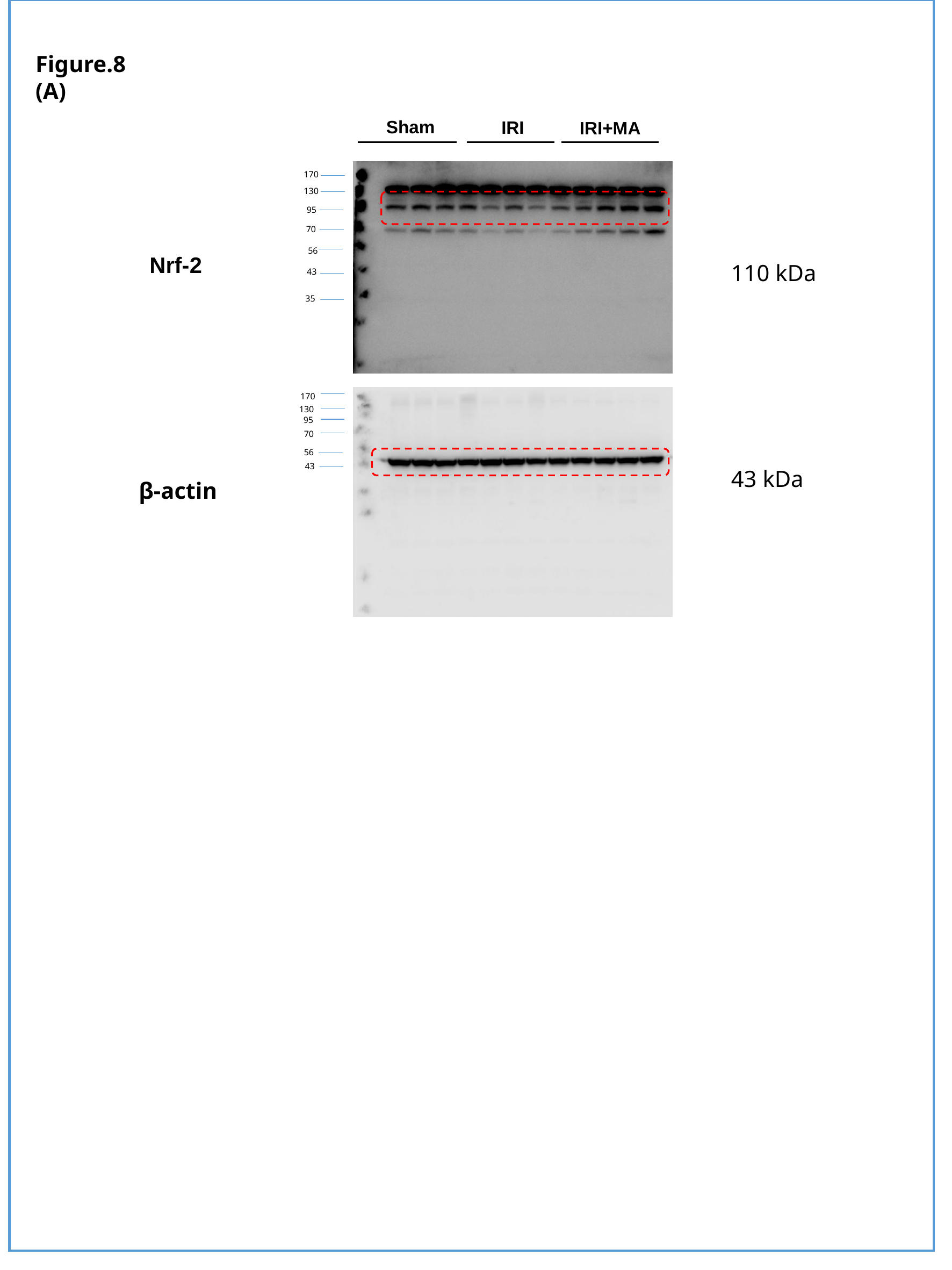

Figure.8 (A)
Sham
IRI
IRI+MA
170
130
95
70
56
43
35
Nrf-2
 110 kDa
170
130
95
70
56
43
 43 kDa
β-actin

## Slide 10
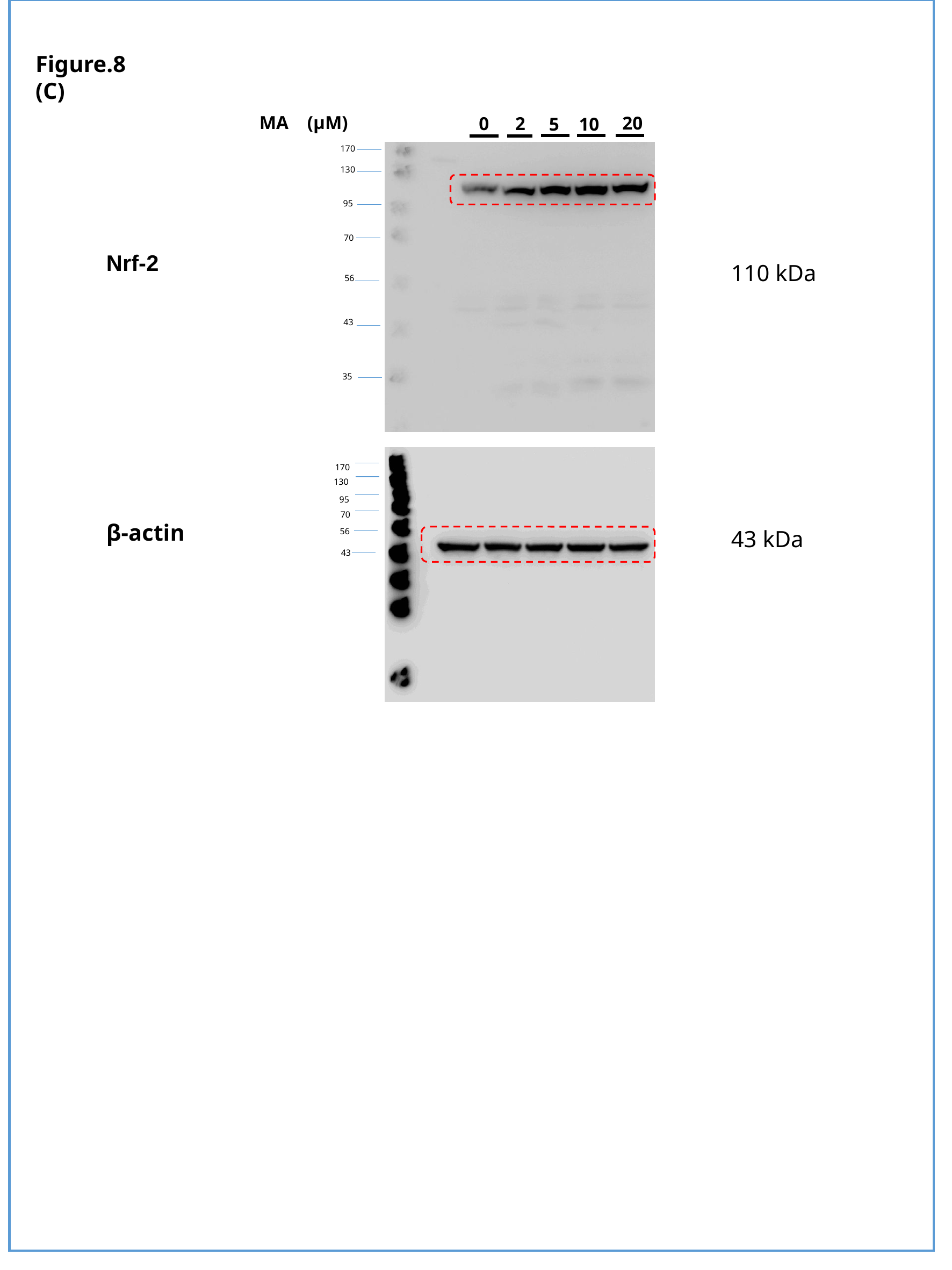

Figure.8 (C)
 MA (µM)
2
10
5
20
0
170
130
95
70
56
43
35
Nrf-2
 110 kDa
170
130
95
70
56
β-actin
 43 kDa
43

## Slide 11
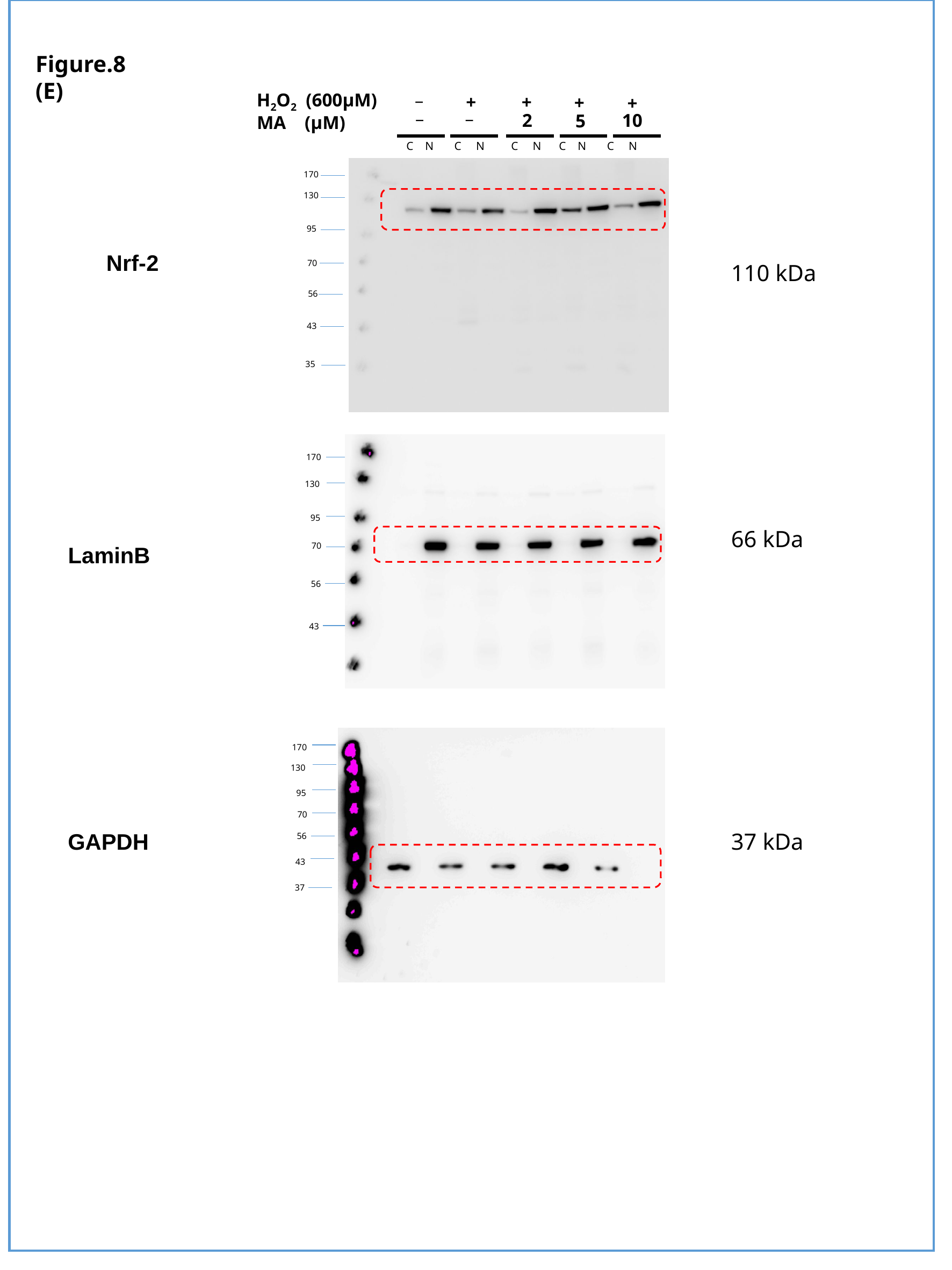

Figure.8 (E)
_
 H2O2 (600µM)
+
+
 +
 +
_
_
2
10
5
 MA (µM)
C N C N C N C N C N
170
130
95
70
56
43
35
Nrf-2
LaminB
GAPDH
 110 kDa
170
130
95
70
56
 66 kDa
43
170
130
95
70
56
 37 kDa
43
37

## Slide 12
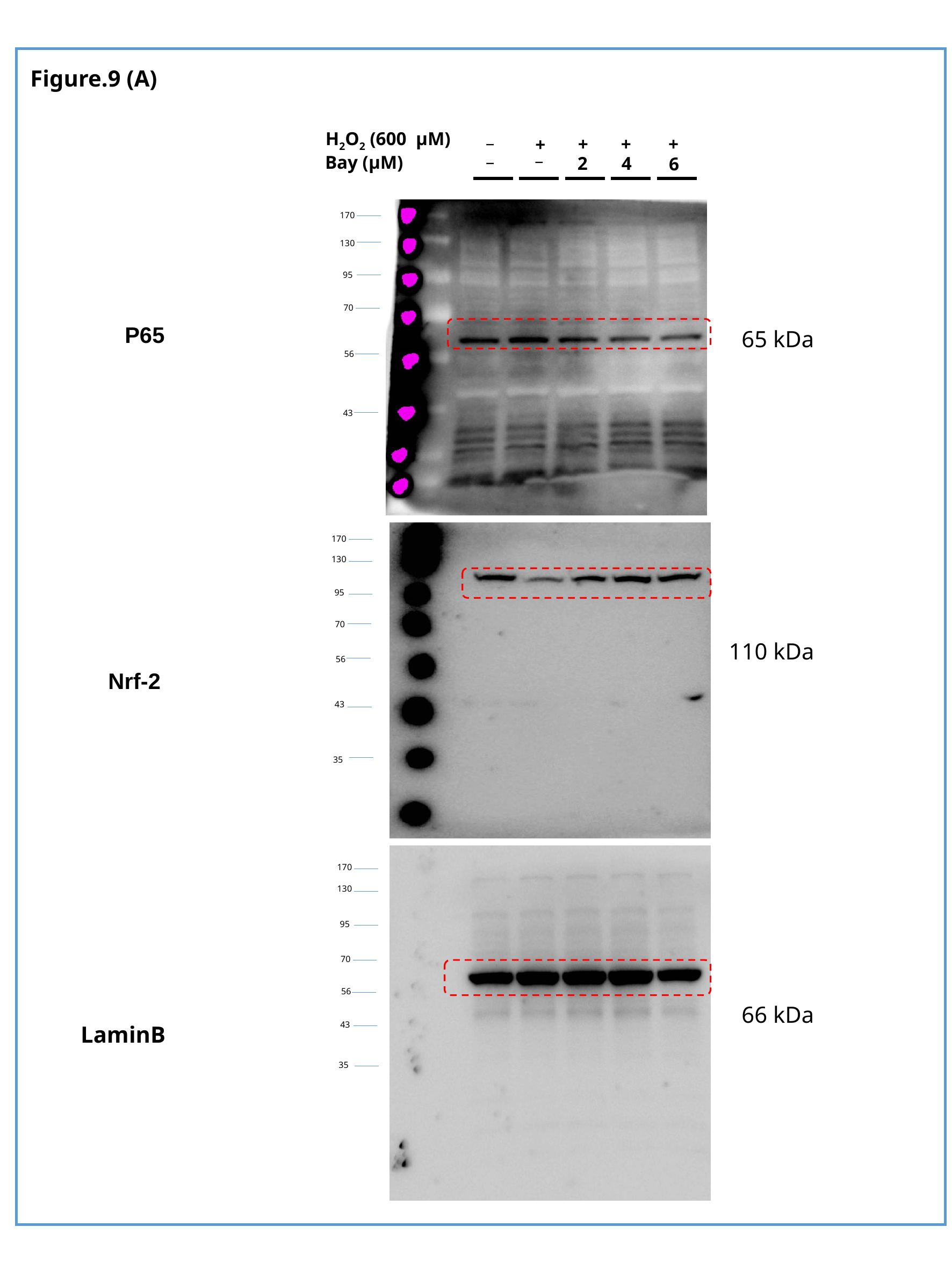

Figure.9 (A)
_
 H2O2 (600 µM)
+
+
 +
+
_
_
 Bay (µM)
2
4
6
170
130
95
70
56
43
P65
 65 kDa
170
130
95
70
56
43
35
 110 kDa
Nrf-2
170
130
95
70
56
43
35
 66 kDa
LaminB

## Slide 13
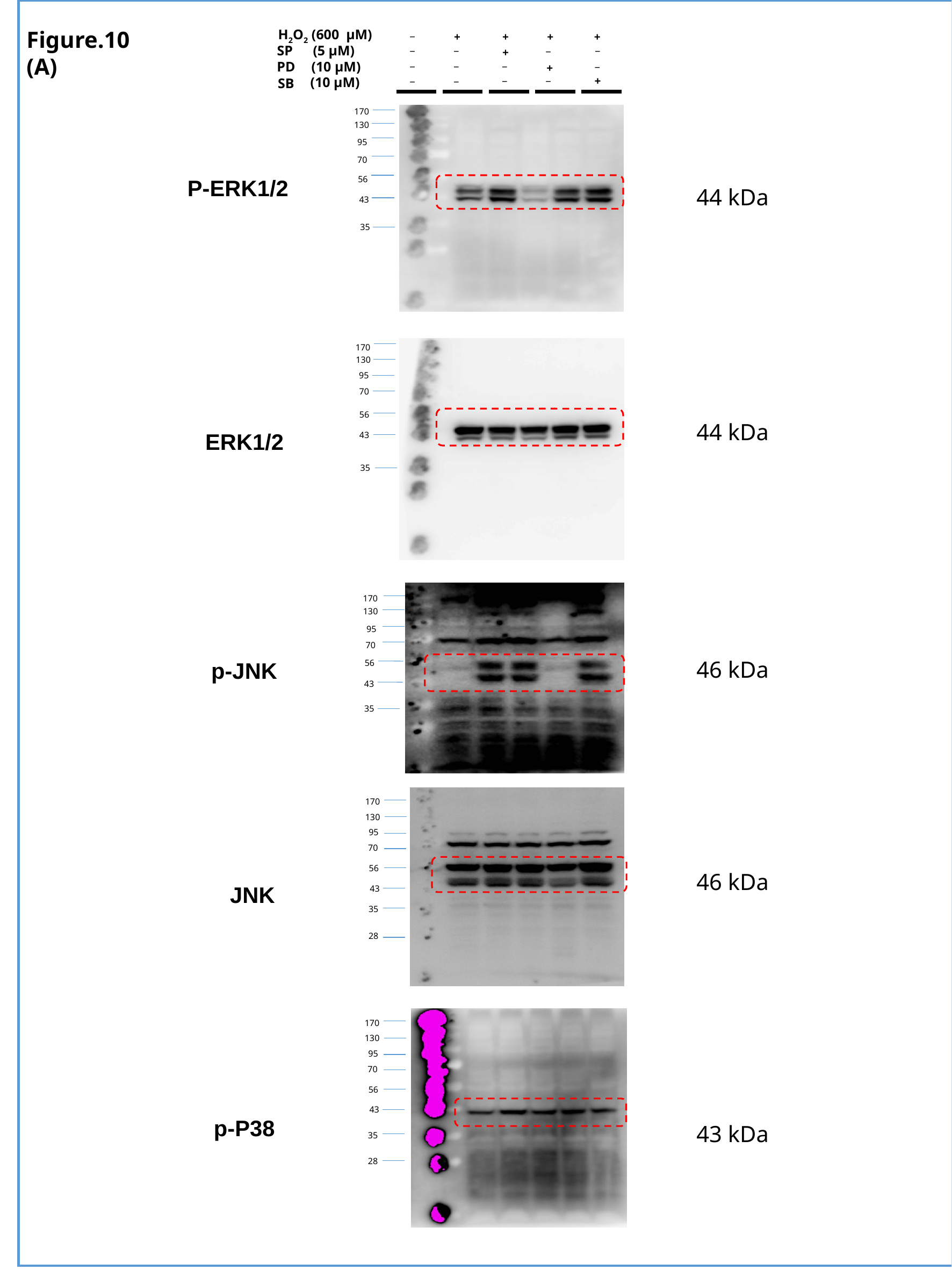

_
 H2O2 (600 µM)
+
+
+
 +
_
_
_
_
 SP
+
_
_
_
_
 PD
+
_
_
_
_
+
 SB
Figure.10 (A)
(5 µM)
(10 µM)
(10 µM)
170
130
95
70
56
43
P-ERK1/2
 ERK1/2
p-JNK
 JNK
p-P38
 44 kDa
 44 kDa
 46 kDa
 46 kDa
 43 kDa
35
170
130
95
70
56
43
35
170
130
95
70
56
43
35
170
130
95
70
 56
43
35
28
170
130
95
70
 56
43
35
28

## Slide 14
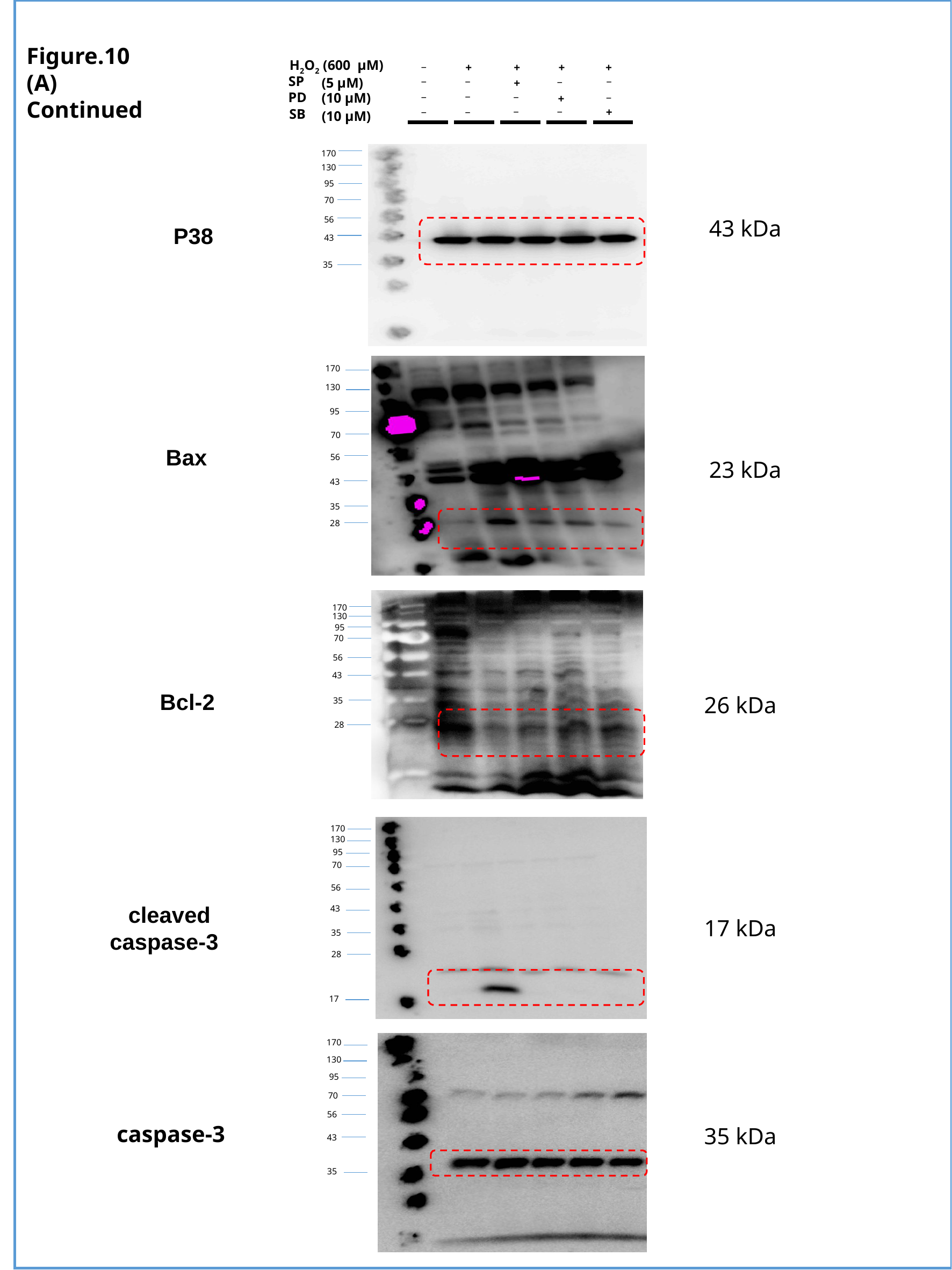

Figure.10 (A)
Continued
_
 H2O2 (600 µM)
+
+
+
 +
_
_
_
_
 SP
+
_
_
_
_
 PD
+
_
_
_
_
+
 SB
(5 µM)
(10 µM)
(10 µM)
170
130
95
70
56
43
35
 43 kDa
P38
170
130
95
70
56
43
Bax
Bcl-2
 cleaved caspase-3
 23 kDa
 26 kDa
 17 kDa
 35 kDa
35
28
170
130
95
70
56
43
35
28
170
130
95
70
56
43
35
28
17
170
130
95
70
56
43
35
caspase-3

## Slide 15
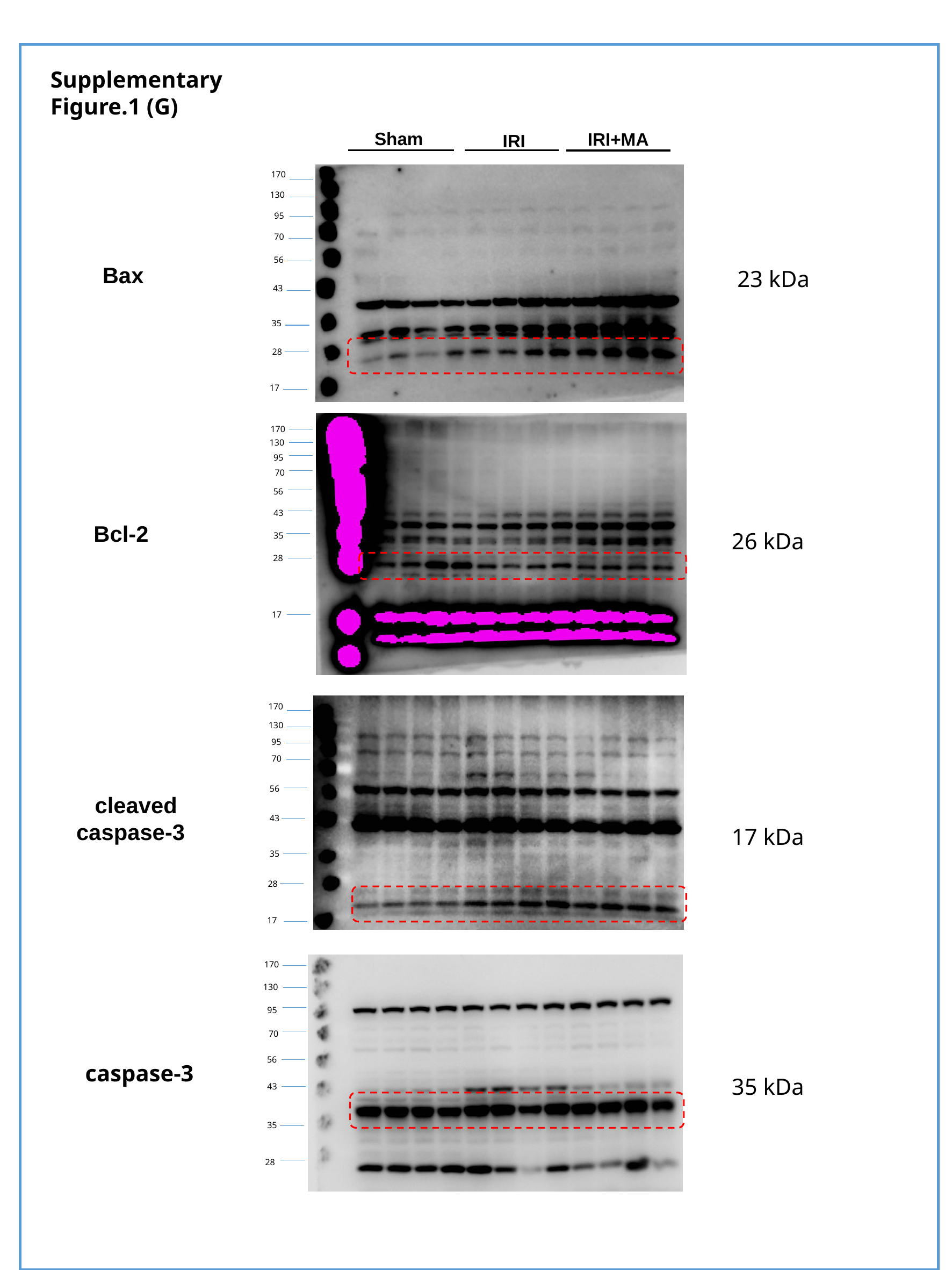

Supplementary
Figure.1 (G)
Sham
IRI+MA
IRI
170
130
95
70
56
43
35
17
Bax
Bcl-2
 23 kDa
 26 kDa
28
170
130
95
70
56
43
35
28
17
170
130
95
70
56
43
35
17
 cleaved caspase-3
caspase-3
 17 kDa
 35 kDa
28
170
130
95
70
56
43
35
28
