# Supplementary figures and images for: Maslinic Acid Attenuates Ischemia/Reperfusion-Induced Acute Kidney Injury by Suppressing Inflammation and Apoptosis Through Inhibiting NF-κB and MAPK Signaling Pathway
Source: Front Pharmacol. 2022 Apr 12;13:807452. doi: 10.3389/fphar.2022.807452 (PMC9039024; doi:10.3389/fphar.2022.807452)

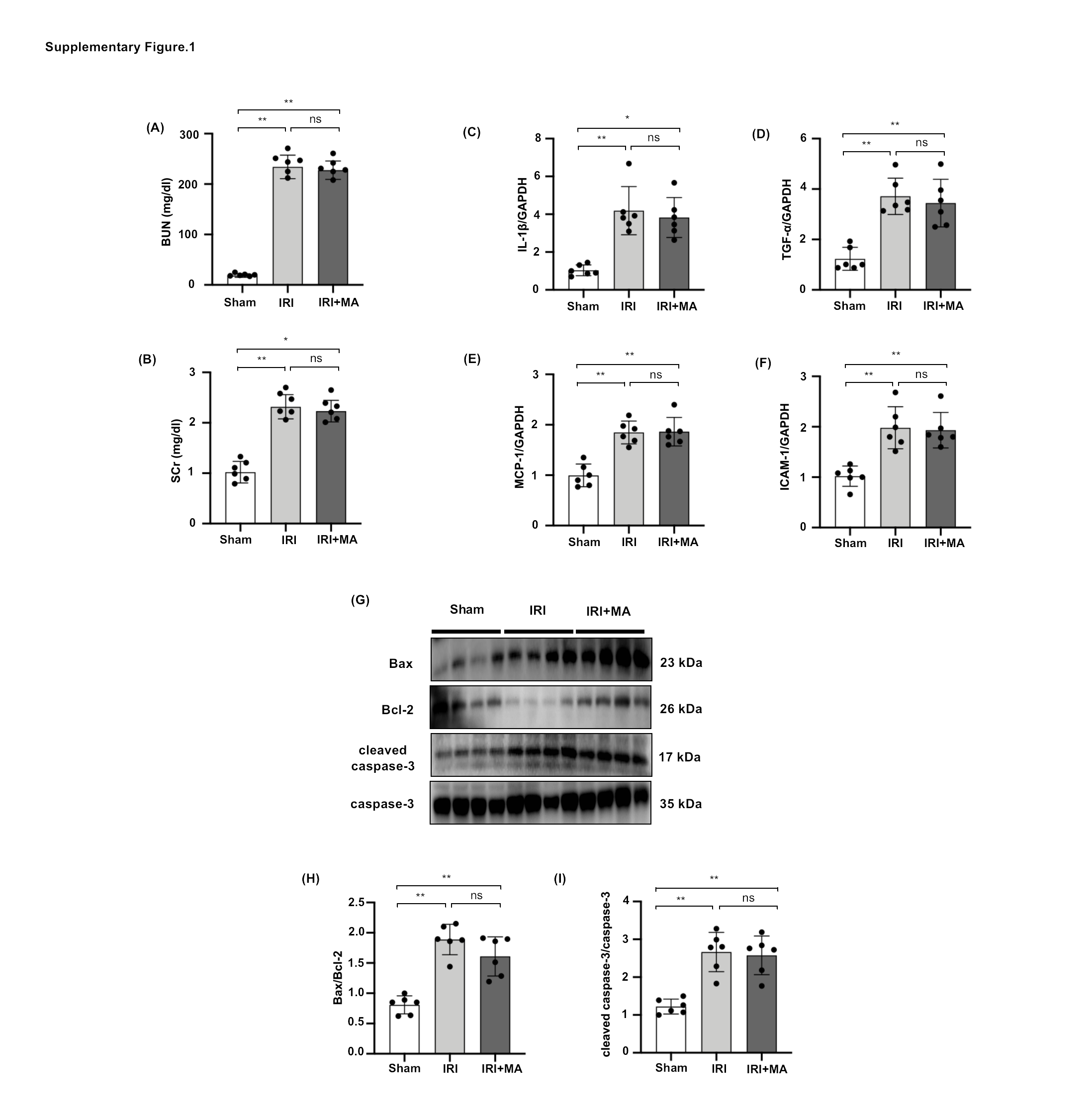

Supplement: Supplementary file 2 [file Image1.TIF]
